# Inferring effective neuronal circuits via network flux counting

**DOI:** 10.1371/journal.pcbi.1014763

**Published:** 2026-09-15

**Authors:** Kevin S. Chen, Ying-Jen Yang

**Affiliations:** 1 Quantitative Biology Institute, Yale University, New Haven, Connecticut, United States of America; 2 Laufer Center of Physical and Quantitative Biology, Stony Brook University, Stony Brook, New York, United States of America; Rutgers University Newark, UNITED STATES OF AMERICA

## Abstract

Extracting circuit mechanisms from neuronal population activity is challenging due to the heterogeneous neuronal properties and diverse strengths in synaptic connections. Standard inference methods, such as Generalized Linear Models (GLMs), typically regress for parameters on all neuronal activity at once. Such a global fitting approach can face identifiability difficulties—for example, where the statistical estimation of strong, opposing weights becomes ill-conditioned in excitatory-inhibitory balanced networks. Here, we introduce FLux-based Effective Coupling (FLEC), a framework that maps spike trains directly to probability fluxes on network state space. Instead of enforcing a single global fit, FLEC infers connectivity and response heterogeneity by quantifying transition rates for each network configuration independently. We demonstrate that FLEC outperforms GLMs and Granger Causality in strongly coupled networks while matching GLM’s performance in standard regimes. Additionally, when combined with Maximum Caliber to construct a minimal dynamical model, the framework better captures temporal statistics—such as inter-spike intervals—than Maximum Entropy models. Robust to parameter variations and unobserved hidden units, and applied to multi-electrode recordings from the salamander retina, FLEC offers a systematic, counting-based tool for inference in nonlinear neuronal circuits.

## Author summary

The brain computes information through a vast network of interacting neurons. To understand these circuits, we need to determine how neurons connect in the network and measure their intrinsic activation properties. However, experiments often provide partial observations of the underlying process: time series of neuronal firing. When a neuron fires frequently, it is difficult to distinguish if it has high intrinsic excitability or if it is receiving strong input from the network. This problem worsens in highly active networks, where strong excitation and inhibition signals cancel each other out and cause standard mathematical methods of inference to fail. To address this, we developed a new statistical tool called FLux-based Effective Coupling (FLEC). Instead of solving complex equations for the entire network all at once, FLEC takes a direct and tractable counting-based approach. It measures how the network transitions between activity states over time and compares counts across different activity levels. This allows FLEC to separate a neuron’s intrinsic excitability from the influence of its neighbors. We demonstrate that FLEC succeeds in regimes where traditional methods break down, providing a more accurate way to infer brain wiring from experimental data.

## Introduction

Uncovering the computational logic of neural circuits requires quantifying the effective synaptic coupling between neurons and their response properties. However, such inference faces two specific hurdles: one must disentangle intrinsic neuronal excitability from the total synaptic input, and simultaneously distinguish the specific weights and types of distinct synaptic sources from that net input signal. An observed high firing rate could arise from strong upstream inputs or simply from high intrinsic excitability. Furthermore, even a fixed aggregate input can present a degeneracy problem: a modest net drive could result from a few weaker excitatory connections or from the cancellation of larger, opposing excitatory and inhibitory currents. This degeneracy is particularly severe in strongly coupled circuits—e.g., in invertebrate systems [1–5] and sensory circuits [6–8]—where strong synaptic inputs saturate neuronal responses and lock the network into strong correlations, thereby obscuring the underlying circuit parameters.

Current circuit inference methods, such as Generalized Linear Models (GLMs) and Granger Causality (GC), provide rigorous probabilistic frameworks that are effective for characterizing receptive fields and functional connectivity [2,6,9–12]. However, stability and identifiability issues can arise in strongly coupled regimes [13,14]. Standard GC, which is based on linear autoregressive processes, struggles to capture the nonlinear spike generation and synaptic interactions inherent to neural circuits [2]. The GLM approach, while explicitly modeling the nonlinear activation of neurons, typically infers couplings by fitting all incoming synaptic weights simultaneously against the neuron’s total aggregated input. Empirically, GLM fails to reliably identify parameters in strongly coupled regimes and may yield solutions that produce unstable activity when simulated [13,14]. In such regimes, including Excitatory-Inhibitory balanced networks, this global fitting procedure encounters a degeneracy problem: large excitatory currents are dynamically canceled by large inhibitory currents. As a result, the regression becomes ill-conditioned, since multiple combinations of large but opposing weights can produce the same net input. Consequently, small estimation errors can lead to large deviations in inferred parameters and introduce systematic bias [14].

To address these limitations without complex parametric assumptions, we introduce the FLux-based Effective Coupling (FLEC) approach. Instead of forcing a global regression, FLEC maps spike trains directly onto trajectories in the network’s configuration state space [15]. By counting transition rates between specific network states, this framework isolates distinct firing configurations—such as a neuron receiving a single input versus multiple concurrent inputs. This allows us to infer specific couplings from a corresponding interaction scenario, thereby avoiding the statistical misidentification caused by a global regression approach. Furthermore, by aggregating these configuration-specific transition rates, FLEC can reconstruct the effective response profile of each neuron across different input levels. This enables the simultaneous recovery of both effective connectivity and heterogeneous response properties directly from statistical counting with minimal parametric assumptions.

In what follows, we demonstrate that FLEC provides a robust, counting-based alternative to regression-based methods. First, using biophysical simulations, we show that FLEC matches GLM performance in weakly coupled E-I balanced motifs but outperforms both GLMs and GC in strongly coupled cases. Second, combined with the principle of Maximum Caliber to construct a minimal dynamical model [16–19], the framework predicts temporal statistics (e.g., inter-spike intervals) more accurately than standard Maximum Entropy models—extending earlier works on neural state space which focused on static codewords (Maximum Entropy) [20,21] by explicitly modeling the transition structure (fluxes) that defines the dynamics. Third, FLEC is robust to different motifs, parameter variations, and unobserved hidden units—making scaling up through a coarse-graining approach possible. We also explore experimental strategies to improve inference in weak coupling regimes with various stimulation protocols, inspired by the “nonequilibrium driving” in [14]. Finally, we apply FLEC to multi-electrode recordings of the salamander retina [22–24] and recover effective motifs that are biologically consistent.

## The FLEC framework

The core idea of FLEC is to shift the perspective from fitting a regression model to counting the probability flow of the system. By utilizing only a *single parameter* for data processing, this framework simultaneously recovers both effective coupling and neuronal heterogeneity directly from the counting statistics. This transformation involves three steps: (1) mapping spike trains to network state transitions, (2) quantifying transition rates, and (3) dissecting these rates to infer network parameters.

### Mapping spiking data to network state transitions

**Fig 1:**
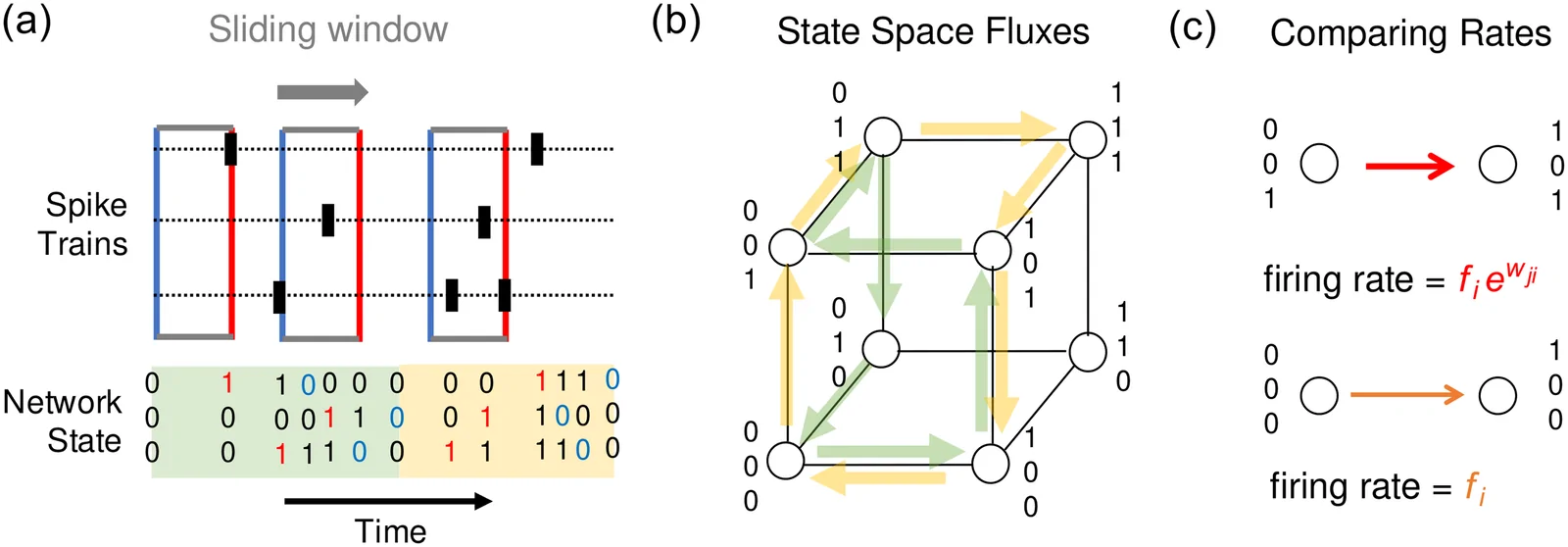
Spiking data can be summarized by flow on the network configuration space. **(a)** Schematic of spike trains converted to a jump process through a sliding window. The spikes enter the window (red) and leave (blue), generating binary events that are converted into the network states shown below. **(b)** Transition of network states shown in a hypercube. The arrows show observed state transitions corresponding to the example in **(a)**. **(c)** Schematic of inferring effective coupling and response function from rates.

First, we consider the configuration space of the network. A network of *N* spiking neurons with binary states can have $2^{N}$ possible configurations—we call them *network states*. For example, three neurons form a cubic network state space with 2^3^ = 8 vertices, as illustrated in Fig 1B. In a continuous-time process, the probability of two neurons changing their state at the exact same instant is negligible. Thus, we follow [15] to consider only *asynchronous transitions*, i.e., a multipartite process [15,25,26]: only one neuron changes state (spikes or falls silent) at a time. This assumption implies that the allowable transitions are restricted to the edges of a hypercube: A network state has *N* possible outgoing transitions, corresponding to any one of the *N* neurons changing its state.

To map spiking dynamics onto jumps on the network state space, we again follow [15] to use a sliding-window method, illustrated in Fig 1A. For state assignment, if a neuron has at least one spike in the window, it is assigned state 1 (active); otherwise, it is 0 (silent). For state transition, we consider two situations as the window slides forward in time. First, when a spike *enters* the window (crosses the red line) and that neuron was previously silent, the neuron updates 0→1. Second, when a spike *leaves* the window (crosses the blue line) and no other spikes from that neuron remain, the neuron updates 1→0. This procedure converts the multivariate spike train into a single trajectory on the hypercube. We choose a window width on the order of ≈10 ms. This timescale is biologically motivated by synaptic biophysics: the postsynaptic potential induced by a neurotransmitter release typically lasts on the order of 10 ms [27,28]. Empirically, time-shifted cross-correlations in retinal spikes also decay significantly outside a 20 ms window [20], suggesting that this is the relevant window for retaining firing information. This window width is the only free parameter in our framework.

### Quantifying transition rates via flux and occupancy

With the population spike train mapped to state space, we can now quantify the system’s dynamics. A naive counting of transitions alone does not identify the coupling strength, since low counts conflate low transition rates with low state occupancy. To obtain the intrinsic *tendency* to transition (the transition rate), we must normalize the flux by the dwell time. For any two connected states x and y, the transition rate $R_{x,y}$ is defined as:


$$\begin{array}{c} R_{x,y}=\frac{\text{total number of transitions }x\mapsto y}{\text{total time spent in state }x}. \end{array}$$ (1)

This set of rates $R_{x,y}$, derived from direct statistical counting, contains all the neuronal excitability and coupling information needed to infer an effective circuit.

### Inferring effective coupling and neuronal response functions

By comparing these conditional firing rates across different network states, we can isolate the contribution of specific neurons (coupling) and the intrinsic properties of the target neuron (heterogeneity). We illustrate this using a 3-neuron motif.

### Spontaneous firing and effective coupling

Consider the transition where neuron 1 spikes, starting from the all-silent state x=(000) to y=(100). The rate of this transition represents the neuron’s *spontaneous firing rate* in the absence of input:


$$\begin{array}{c} f_{1}=R_{\left(000),(100\right)}. \end{array}$$ (2)

Now, consider the case where only neuron 3 spikes, x=(001). The rate at which neuron 1 spikes in this context is *R*_(001),(101)_. As illustrated by Fig 1C, comparing this driven rate to the spontaneous rate reveals the *effective coupling w*_3,1_ from neuron 3 to neuron 1. We define the coupling strength as the natural logarithmic gain:


$$\begin{array}{c} w_{3,1}=\ln\frac{R_{\left(001),(101\right)}}{R_{\left(000),(100\right)}}. \end{array}$$ (3)

The logarithm mapping ensures that a rate increase (decrease) reflects a positive (negative) coupling. This definition echoes the notion of affinity in chemistry and nonequilibrium physics [29,30] and requires no optimization or data-dependent choices of basis functions.

### Inferring neuronal heterogeneity via response functions

Because the network transition rates $R_{x,y}$ in FLEC consist of firing rates under all configurations, we can further infer the effective *response function* $\varphi_{i}$ for each neuron by treating the inferred $w_{j,i}$ as synaptic “current-like” input. To illustrate, we again focus on neuron 1 in the three-neuron motif. Here, the transition rates contain four data points that determine the shape of the response function $\varphi_{1}(\cdot )$:


$$\begin{array}{cc} R_{\left(000),(100\right)} & =\varphi_{1}(0)=f_{1}\\ R_{\left(010),(110\right)} & =\varphi_{1}(w_{2,1})\\ R_{\left(001),(101\right)} & =\varphi_{1}(w_{3,1})\\ R_{\left(011),(111\right)} & =\varphi_{1}(w_{2,1}+w_{3,1}). \end{array}$$ (4)

**Fig 2:**
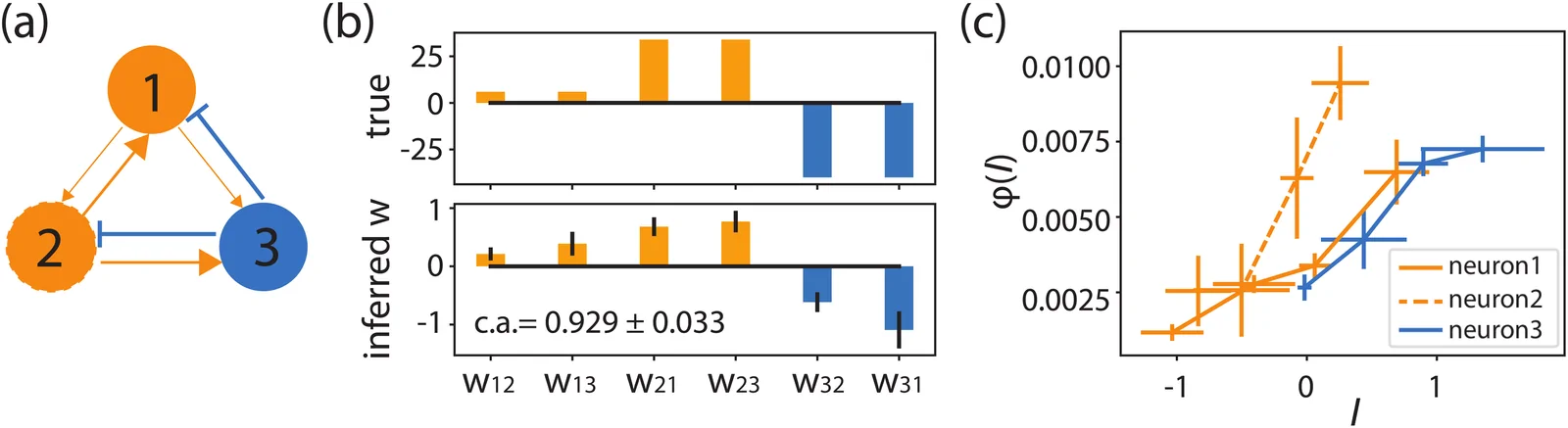
FLEC simultaneously recovers effective coupling and neuronal heterogeneity in an E-I balanced circuit. **(a)** Schematic of the ground-truth LIF circuit. **(b)** Comparison of true synaptic weights (top) and inferred effective couplings (bottom). Error bars indicate standard deviation across 10 trials. **(c)** Reconstructed response functions φ(I). The x-axis *I* represents the total effective synaptic input (sum of active *w*’s). FLEC correctly identifies that Neuron 2 has a lower biophysical threshold (left-shifted curve) compared to Neurons 1 and 3.

By plotting these rates against their corresponding effective inputs ($0,w_{2,1},w_{3,1},w_{2,1}+w_{3,1}$), we can reconstruct the nonlinear response curve for neuron 1. Neuronal heterogeneity in excitability can thus be inferred by repeating this construction for each neuron. An example is shown in Fig 2.

## Results: FLEC provides robust inference for Spiking Motifs

We validate FLEC using biophysical simulations of Leaky-Integrate-and-Fire (LIF) networks. To test the framework’s limits, we focus on the strong coupling regime (high signal-to-noise ratio), a condition common in sensory circuits [7,8,24] and invertebrate systems [1–4] but difficult for standard inference methods [14]. We systematically scan across parameters to ensure the results are representative (Figs C and D in S1 File).

### Simultaneous inference of structure, heterogeneity, and causality

We first test FLEC on a fully connected E-I balanced motif, a regime where strong excitation and inhibition cancel out to produce fluctuation-driven spiking (Fig 2A). Despite strong cancellation of opposite signals, FLEC effectively identifies the synaptic architecture. As shown in Fig 2B, the inferred effective couplings ($w_{ij}$) correctly capture *both* the sign (excitation vs. inhibition) and the relative magnitude of the ground-truth synaptic weights. We quantify this accuracy using the cosine angle between the inferred and true weight vectors:


$$\begin{array}{c} \text{cosine angle}=\frac{w_{infer}\cdot w_{truth}}{\left||w_{infer}||\cdot ||w_{truth}|\right|}. \end{array}$$ (5)

This strict metric confirms that the inference recovers the type and relative strength of connections, not just the binary network topology.

Crucially, FLEC disentangles neuronal heterogeneity from synaptic drive. In the simulated data, Neuron 2 was set to have a lower biophysical firing threshold (-60 mV) than Neurons 1 and 3 (-50 mV). A standard regression model might mistake this high intrinsic excitability for strong external input (e.g., a larger inferred *w*_12_). FLEC, however, reconstructs the effective response function φ(I) for each neuron by aggregating rates across different input configurations. As shown in Fig 2C, the inferred response curve for Neuron 2 is distinctly shifted to the left (higher excitability), while Neurons 1 and 3 overlap. This demonstrates that FLEC separates synaptic contributions from intrinsic biophysics without prior assumptions about the neuron model.

**Fig 3:**
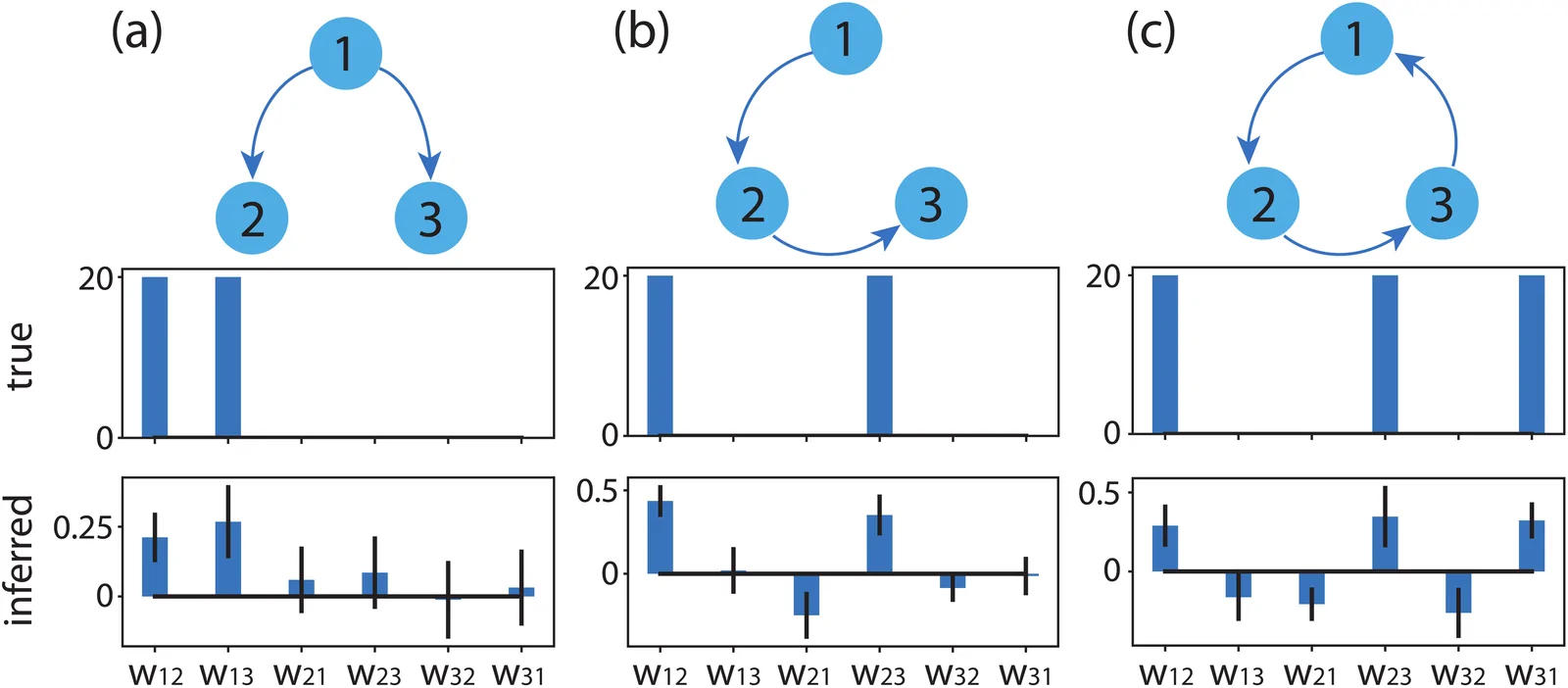
Distinguishing causality from correlation in distinct circuit motifs. **(a)** Common Input: FLEC correctly avoids inferring spurious links between correlated neurons 2 and 3. **(b)** Chain: FLEC identifies direct connections and ignores indirect correlations (1 to 3). **(c)** Cyclic: FLEC captures the directionality of feedback loops.

Finally, we verify that FLEC distinguishes direct causality from indirect correlation by testing three canonical motifs (Fig 3):

1. Common Input (Fig 3A): Neurons 2 and 3 are highly correlated due to the shared input from Neuron 1. FLEC correctly infers, within the error bar, zero coupling between them, avoiding the strong spurious links often found by correlation-based methods.
2. Chain (Fig 3B): Neuron 1 drives 2, which drives 3. FLEC correctly identifies the direct feedforward chain. While weak spurious inhibitory links appear in the reverse direction (likely due to refractory effects appearing as inhibition), the dominant causal structure is preserved.
3. Cyclic Loop (Fig 3C): FLEC captures the directionality of the feedback loop even in the presence of complex recurrent dynamics.

Together, these results demonstrate FLEC’s capacity to resolve the precise origins of spiking activity—distinguishing intrinsic biophysics from synaptic drive, and direct causal links from network-wide correlations.

### Superior performance in strongly coupled regimes

To benchmark FLEC against established standards, we compare its structural inference performance with two widely used frameworks: Generalized Linear Models (GLMs) and Granger Causality (GC). GLMs typically model the spike train as a Poisson process where the instantaneous firing rate is determined by a linear summation of filtered inputs and spike history, followed by a static nonlinear function (e.g., exponential) converting the total input to a rate [6,10,14,31]. While powerful, this Linear-Nonlinear assumption can become inaccurate when synaptic interactions are strong enough to saturate the nonlinearity (see Methods for GLM implementation details). On the other hand, GC tests for directed functional connectivity based on prediction improvement, typically using a linear autoregressive framework to determine whether the past history of a source neuron predicts the future of a target neuron [32]. We also compare a GC measure for spike trains (spkGC), which is similar to GC but formulated for point process models [33].

**Fig 4:**
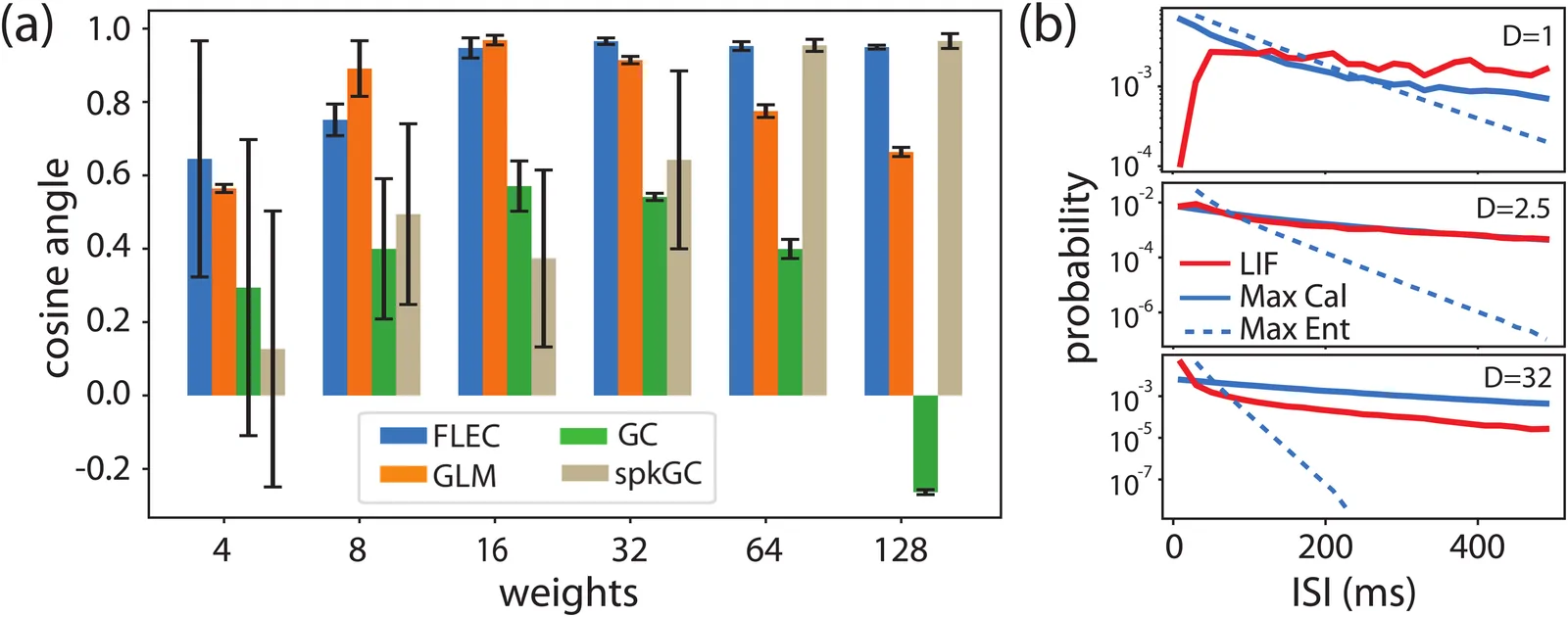
Comparison of network inference and spike train reconstruction. **(a)** The cosine angle between inferred and true weights as a function of the network strength in the E-I balanced motif. The weights here are scalars *W* so that in each case the final weights are $WW_{ij}$, where $W_{ij}$ is the E-I balanced motif with unit excitatory connection. The four colors indicate different methods: FLEC proposed in this work, Granger causality (GC) [2], generalized linear model (GLM) [6], and spiking GC (spkGC) [33]. Error bars show std across three instantiations. **(b)** Example ISI distribution of the E-I balanced motif, with three different noise strengths *D* selected. The empirical measurement from LIF spike train, Max Cal reconstruction from the fitted FLEC model, and Max Ent prediction are shown.

We systematically evaluate these methods alongside FLEC on simulated data across a range of synaptic weights (Fig 4; see Appendix A in S1 File for the discussion of varying other simulation parameters). As shown in Fig 4A, in the weak coupling regime, FLEC yields performance comparable to GLMs and spkGC in certain regimes, while GC performs poorly, as expected given its linear assumption on binary spike data. A distinct divergence occurs in the strong coupling regime (weights > 15, comparable to the gap between resting potential and threshold potentials). Here, GLM performance degrades, likely due to ill-conditioned regression where the linear sum assumption struggles to resolve the cancellation between strong excitation and inhibition [14]. In contrast, FLEC maintains high accuracy (cosine angle > 0.85, matching spkGC’s good performance) throughout the strong coupling regime, demonstrating robustness where GLM fails.

Beyond structural connectivity, a comprehensive inference framework should also capture the system’s dynamical statistics. We tackle this by reconstructing the Inter-Spike Interval (ISI) distribution. By combining FLEC-inferred transition rates with the principle of Maximum Caliber (Max Cal), we construct a minimal dynamical model based directly on the measured transition rates from counting—a continuous-time Markov Jump Process—and compare it against a standard Maximum Entropy (Max Ent) model [20,21,34,35].

As shown in Fig 4B, the standard Max Ent model predicts a geometric-like ISI distribution. This is because Max Ent, constrained only by static firing rates and synchronous correlations, treats network states as independent snapshots—effectively rolling a biased die at every time step without memory. In contrast, the FLEC-derived MJP incorporates probability fluxes, explicitly constraining the transitions between states. This allows the model to capture the dynamical structure of the trajectory, providing a correction to the ISI prediction that matches the ground-truth LIF simulations more closely than Max Ent across different noise levels (*D*) (see Appendix A in S1 File for more discussion on how noise strengths affect the ISI inference).

Collectively, these comparisons demonstrate that FLEC not only recovers the physical coupling strength where regression fails but also captures the essential temporal statistics missed by static Ising-like models.

### Scalability via coarse-graining and robustness to hidden units

Inspired by successful inference in small motifs, we seek to apply FLEC to larger networks. However, a scaling argument reveals the challenge of a direct application. Consider a spiking network of *N* neurons, each modeled by a binary state. The network has $2^{N}$ possible configurations, and under the *multipartite* assumption that only one change happens at an instant in continuous time, there are $N2^{N}$ transitions in the state space. Analytical FLEC requires counting fluxes on the $N2^{N}$ transitions and the dwelling times of the $2^{N}$ configurations. The dimensionality and the requirement of data grow exponentially: there are about 10^4^ transitions when *N* = 10, which grows to 10^32^ when *N* = 100. In Fig G in S1 File, we show that empirically each transition requires at least 100 transition counts for the inference performance to saturate. Therefore, a perturbative coarse-grained approach is needed to go beyond motifs.

Neural recordings typically sample only a subset of a circuit, leaving many interactions unobserved. FLEC addresses this partial observability through coarse-graining. We define the marginal effective coupling $g_{ij}$ simply by counting the flux and dwell times restricted to the state space of the neuron pair (*i*, *j*), ignoring all others. Theoretically, this $g_{ij}$ relates to the higher-order conditional couplings (*w*) through marginalization (see Eqs. 1 and 2 in S1 File). Physically, $g_{ij}$ captures the marginal influence from neuron *i* to *j*, absorbing all indirect pathways mediated by the rest of the network. In contrast, $w_{ij}$ resolves the interaction conditioned on the observed neighbors (e.g., the third neuron in a triplet). Comparing *g* and *w* thus allows us to filter out indirect correlations mediated within the observed group, distinguishing local causality from correlation.

**Fig 5:**
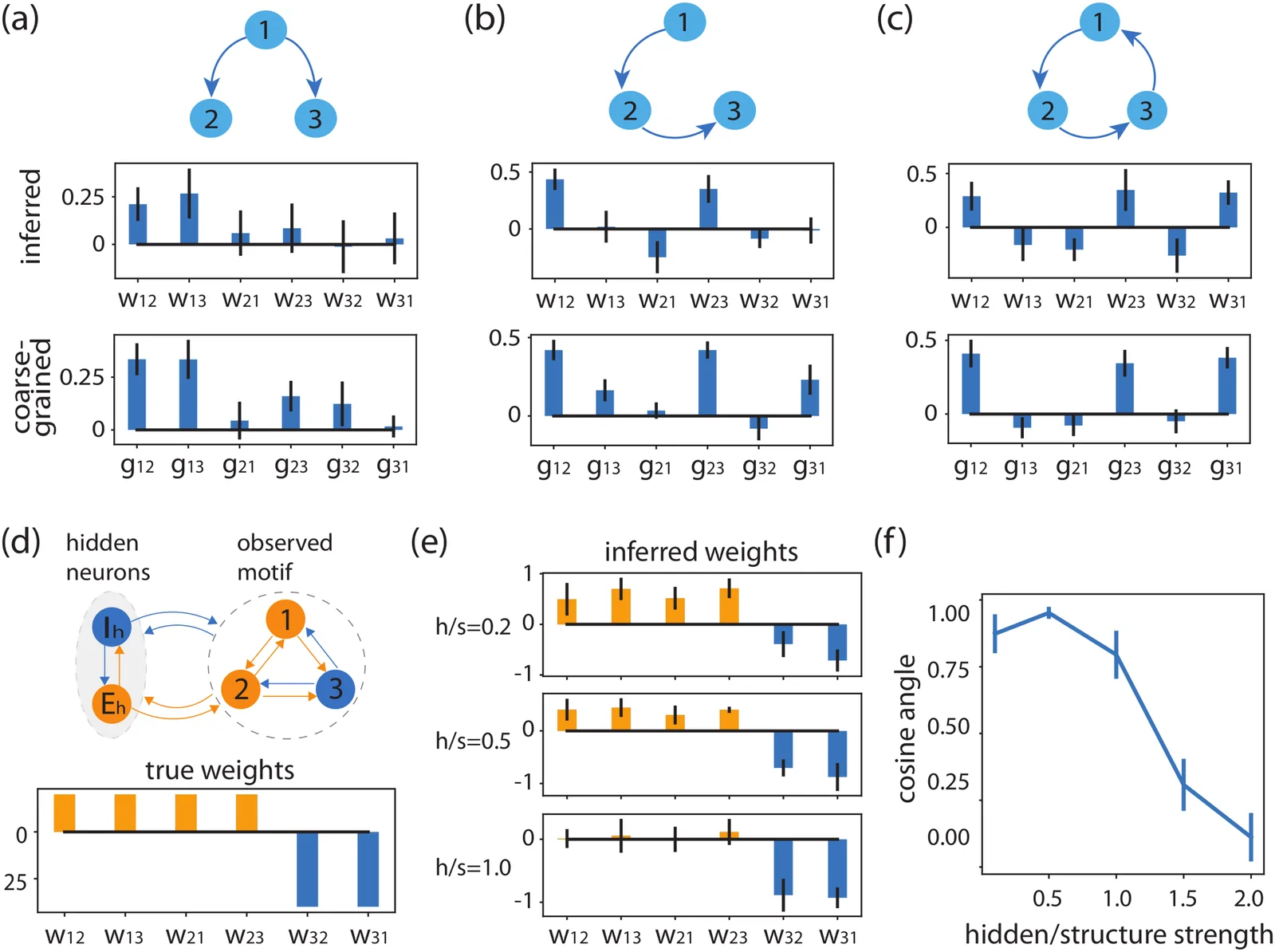
Coarse-graining validates robustness across motifs and against latent interactions. **(a-c)** Comparison of inferred conditional weights (*w*, full model) and marginal weights (*g*, pairwise model) for different motifs. **(d)** Schematic of subsampling: a 3-neuron motif is observed while embedded in a larger 5-neuron network. **(e)** Inference accuracy of the observed motif as a function of hidden-to-structure connection strength (*h*/*s*). **(f)** The performance decays slowly, retaining accuracy even when hidden inputs are comparable to observed weights.

Comparing marginal *g* and conditional *w* weights offers a perturbative approach to diagnose network topology and filter spurious connections (Fig 5A):

- **Common Input (Correlation vs. Causality):** In a motif where 1 drives 2 and 3, the pairwise inference shows strong couplings *g*_23_ and *g*_32_ due to correlation. However, upon conditioning on neuron 1, the triplet couplings *w*_23_ and *w*_32_ vanish, correctly revealing no direct link.
- **Chain (Indirect Pathways):** In a 1→2→3 chain, the marginal *g*_13_ is strong, capturing the indirect flow. Accounting for the intermediate neuron 2 causes *w*_13_ to vanish, successfully distin*g*uishing the direct chain from a feedforward shortcut.
- **Network Modulation and Indirect Pathways:** Discrepancies between the marginal *g* and the conditional *w* can suggest the presence of indirect pathways or network modulation. Intuitively, *w* is a correction to *g* by conditioning the transition statistics on the third neuron being silent. In Appendix C in S1 File, we show that g≈w only in the low firing rate limit of the third neuron. Since *g* generally mar*g*inalizes out all influence of the unobserved network, comparing it against the basal conditioned coupling *w* and the driven coupling $w^{\prime }$ (computed the same way as *w* but when the third neuron is active) provides quantitative clues on how latent network activity modulates the pairwise correlation.

This implies that for large networks, one can *hierarchically distinguish direct connections* from indirect ones by expanding the conditioning set (pair → triplet →⋯).

We quantify the limits of this local inference by subsampling a larger simulated network. We construct a 5-neuron LIF circuit but restrict the partial observations to a 3-neuron subset, treating the remaining two as hidden units (Fig 5D). We find that the inference quality of the observed motif decays gradually as the hidden input strength increases (Fig 5F). FLEC correctly identifies the signs and relative strengths of the observed motif even when the hidden interactions are roughly half the strength of the observed ones (h/s≈0.5). This robustness demonstrates that FLEC can extract meaningful local circuitry without requiring a fully observed system.

**Fig 6:**
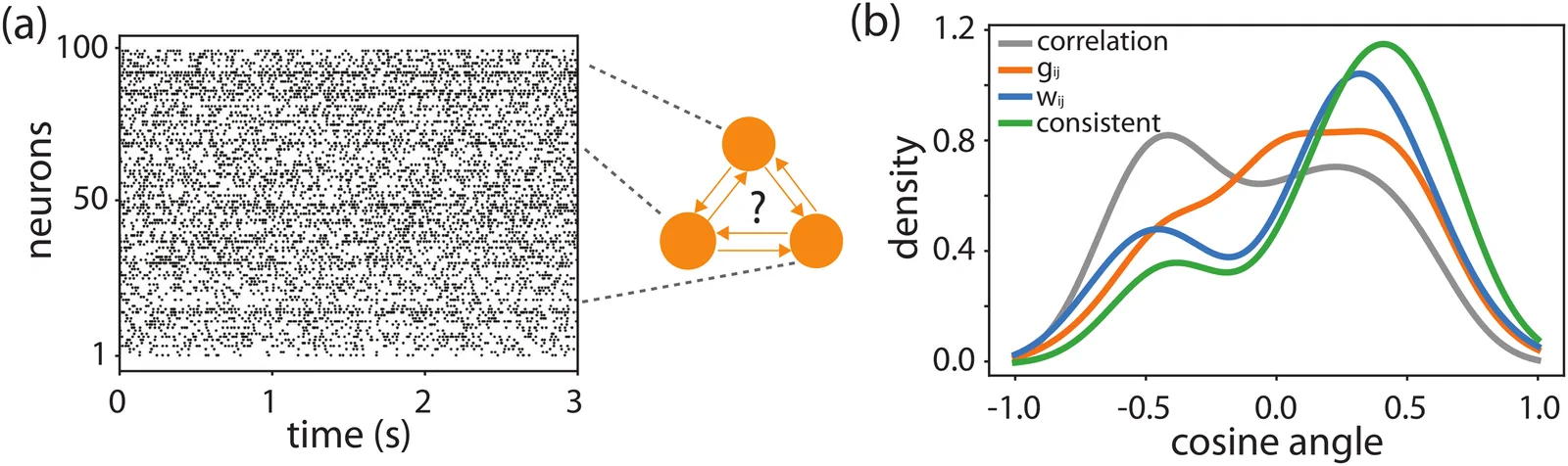
Scaling FLEC to large spiking networks. **(a)** An example time series of a random E-I network with 100 neurons. From this neural population we subsample pairs ($g_{ij}$) or triplets ($w_{ij}$) to infer effective couplings. **(b)** Comparing the cosine angle between inferred connectivity and ground truth for different inference settings. Correlation refers to the instantaneous spike train correlation. FLEC with pairs ($g_{ij}$) and triplets ($w_{ij}$) are shown. Consistency is for pairs where the sign inferred from pairs and triplets agree.

Motivated by the results from coarse-graining and conditions with latent interactions, we further demonstrate an application to a larger simulated 100-neuron spiking network (Fig 6). Compared to raw spike train correlations, applying FLEC to subsampled motifs systematically improves the overall inference, shifting the cosine angle distribution closer to the ground truth. Furthermore, when evaluating purely for sign inference on edges where the pairwise and triplet models agree, FLEC correctly predicts the sign for 36% of the actual excitatory connections and 91% of the inhibitory connections. This asymmetry—below chance for excitatory edges, above chance for inhibitory ones—reflects the same systematic bias toward negative effective couplings noted above, rather than an absence of excitatory signal. Correct inference can be further boosted by comparing the consistency between the marginal ($g_{ij}$) and conditional ($w_{ij}$) weights.

**Fig 7:**
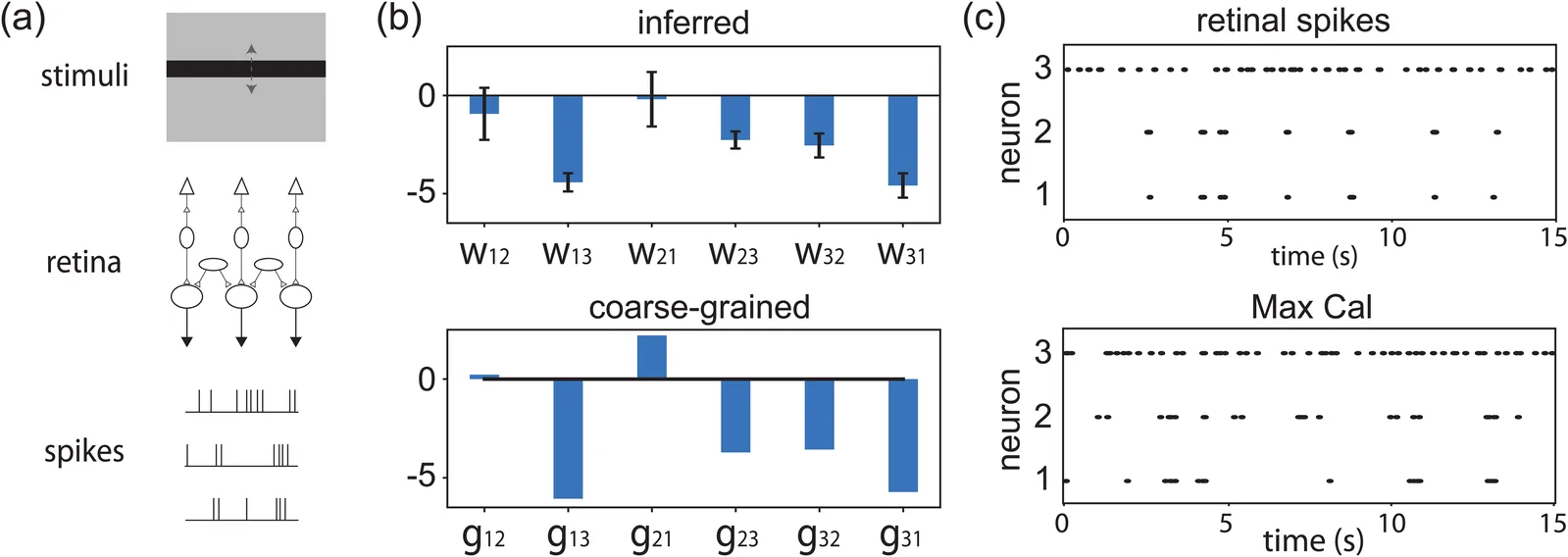
Inference applies to functional connectivity in the salamander retina. **(a)** Schematic of RGC spike trains recorded under stochastic visual stimulation. **(b)** Inferred effective circuit showing lateral inhibition and shared excitation. The top panel shows mean and standard deviation for coupling pairs while sampling across different conditional neurons (*w*, triplet models) across the recording (*N* = 53). For example, the error bar in *w*_12_ indicates the variation of inferring the same coupling 1↦2 across different chosen “neuron 3.” In contrast, the error bar in *w*_23_ shows the variation of its value for the original 2↦3 across different chosen “neuron 1”. The full inference result is shown in Fig H in S1 File. The bottom panel shows the marginal weights (*g*, pairwise models). The consistency between the conditional model (*w*) and the coarse-grained marginal model (*g*) supports the robustness of the inferred functional couplin*g* against potential latent inputs. **(c)** The measured spike train (top) and Max Cal reconstruction from the fitted FLEC model (bottom). We show additional retinal motif examples and discuss effects of time window in Figs E and F in S1 File.

We further explore the application of FLEC to biological measurements, such as the retinal spike train. The data are from multi-electrode recordings of salamander retinal ganglion cells (RGCs). Different from previous simulation work, we note that here the ground truth connectivity is absent and the reported result should be viewed as an effective interaction captured by FLEC. We choose a triplet motif out of the overall network for inference (Fig 7). The inference reveals a motif characterized by strong lateral inhibition and weaker mutual excitation, consistent with RGC physiology [23,24]. The neural mechanism of lateral inhibition likely comes from the amacrine cells that carry inhibitory projections in the inner plexiform layer, which modulate parallel processing bipolar inputs and signaling between RGCs [36,37]. The weak mutual excitation may come from dendritic gap junctions between RGCs [38,39].

The overall consistency between the conditional couplings (*w*) and the marginal couplings (*g*) in this RGC triplet suggests that the inferred links are robust features of the local circuit rather than artifacts of unobserved latent variables. Beyond comparing the inferred marginal couplings with the conditional, we can also test the robustness of the latter by changing the conditional neurons. For example, for inferring the effective coupling from the original neurons 1–2, we compute different *w*_12_ by choosing “different conditional neuron 3.” Similarly, for inferring the original 2↦3, we compute *w*_23_ by choosing “different conditional neuron 1.” We find that the inference for weak or positive coupling is more variable, whereas the inferred inhibitory coupling is robust across different selected conditional neurons (Fig 7B). This systematic bias towards identifying negative weights is consistent with our findings in the 100-neuron network test reported above.

In the retinal spike train, activity is sparse and the signal can be heavily modulated by shared external visual inputs [20,22] (Fig 7C). While the lack of stimulus state labels and cell-type classifications in the data precludes detailed anatomical analysis, the statistical signatures recovered by FLEC offer a phenomenological interpretation. Across the sampled triplets, the basal conditional couplings ($w_{ij}$, computed when the third neuron is silent) are predominantly negative, suggesting widespread effective inhibition or refractoriness during baseline activity. Conversely, the driven couplings ($w_{ij}^{\prime }$) frequently shift toward positive values (panel B of Fig H in S1 File). We interpret this shift as an indication that the activation of the third neuron acts as a statistical proxy for the presence of a spatio-temporally correlated visual stimulus. The increment of effective coupling from *w* to $w^{\prime }$ could be due to an unobserved common drive that simultaneously excites the recorded neurons, transiently overcoming the baseline effective inhibition to generate highly correlated spiking. Thus, rather than claiming a definitive anatomical map, these results show that FLEC could provide statistical markers that reflect baseline network suppression from stimulus-induced correlations.

We have also studied the effective window effects, which may alter how such time-varying input is integrated, and provided discussion in Appendix B in S1 File. Together, these results show that through coarse-graining FLEC can be applied to larger systems with hidden units and reveal consistent, robust inference performance. These aspects open future application to realistic biological measurements.

## Summary and discussion

We presented Flux-based Effective Coupling (FLEC), a method that infers circuit parameters by counting transitions in the network state space rather than fitting global regression models. By isolating state configurations, FLEC disentangles intrinsic excitability from synaptic drive and resolves causal structure in strongly coupled regimes (e.g., E-I balanced), where regression-based methods (GLM, GC) often encounter ill-conditioned estimation. Additionally, when combined with Maximum Caliber, the inferred transition rates yield a minimal Markov Jump Process that predicts temporal statistics, such as inter-spike intervals.

Since neural recordings are typically partial observations of circuits in a larger network, inference remains necessarily local. Our results on coarse-graining demonstrate that comparing marginal couplings (*g*, pairwise) with conditional couplings (*w*, triplet) identifies indirect pathways (Fig 5). Moreover, the subsampling analysis confirms that FLEC degrades gradually when significant inputs remain unobserved (Fig 5). These results support a hierarchical strategy for large networks: initialize inference with local motifs, then expand conditional analyses to refine the circuit map as the number of recorded neurons increases. We demonstrate a proof-of-principle application on a simulated 100-neuron spiking network (Fig 6). We leave a comprehensive exploration of such hierarchical inference to future studies.

We note limitations inherent to statistical inference of network properties from time series data. First, FLEC extracts *effective* connectivity—the net causal influence—which does not strictly map to anatomical synapses [6,14,40]. Constraining flux counting with structural priors (e.g., from connectomics) remains an avenue for future work. Second, the method relies on sufficient transition statistics. The network state space grows exponentially with the number of neurons [15,21,41], meaning that rarer transitions become harder to sample. While we observed robustness with finite data (Fig G in S1 File), this sets a practical limit on the size of the motif that can be inferred simultaneously. Consequently, in practice, *users must strike a balance*: one can either analyze smaller, coarse-grained motifs with high statistical confidence, or attempt larger networks while accepting higher sampling sparsity [41].

While the application of FLEC to retinal spike train yields consistent inference results, we cannot exclude the effect from dynamics in the visual stimuli presented during the experiments. Despite this caveat, our result captures the sparse code with parallel information in the retina [42,43], realized by mutual inhibition in the small time windows. Even though the retinal population is driven by spatially correlated stimuli, the effective connectivity shows a certain degree of mutual inhibition that may decorrelate signal [42]. This is reminiscent of an alternative analysis of the retinal spike train, where results show that the irreversibility of spiking patterns is not affected by different stimulus statistics [15]. Interestingly, we find that positive weights can be inferred when the conditional triplet neuron has higher firing rate (Fig H in S1 File) or larger window size to include firing patterns (Fig D in S1 File). Indirectly, these effects are similar to having stronger external input to the system to increase overall firing rate, thereby revealing non-negative interactions. If the stimulus information is available in experimental measurements, a better approach is to treat the stimulus as a layer of stimulating neurons with labels indicating its on or off periods and include them into FLEC. This modification will help generalize the FLEC method to input-driven systems in the future.

**Fig 8:**
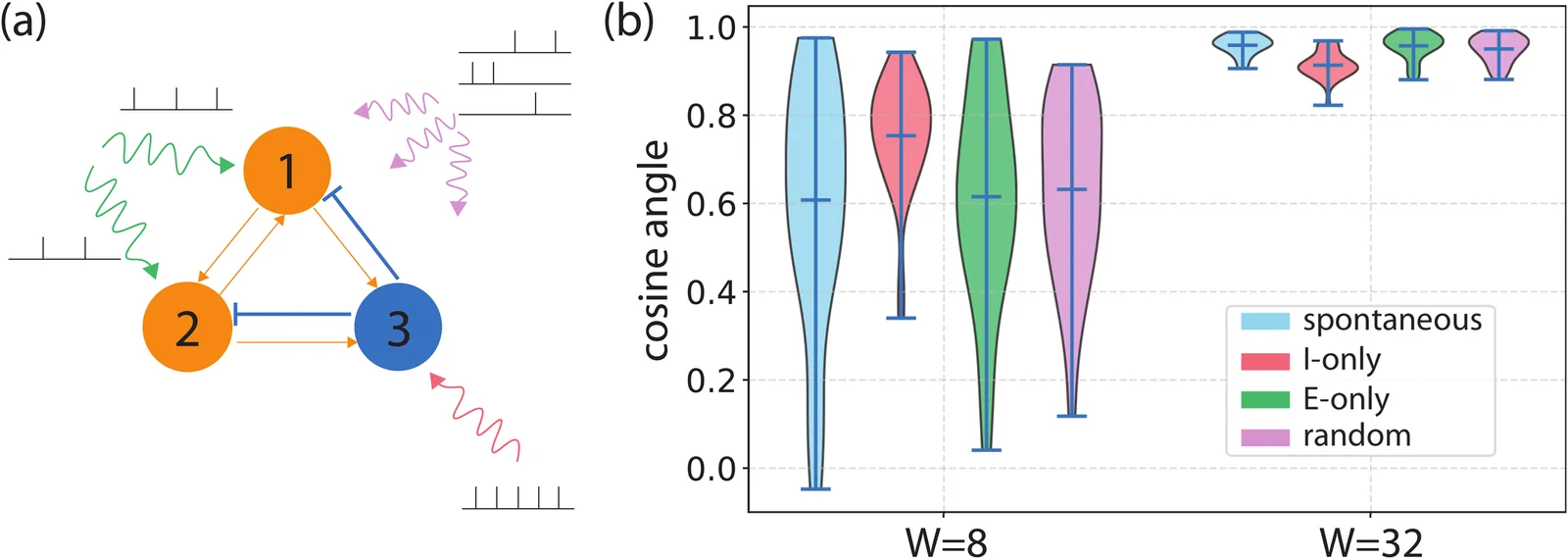
Potential remedy for inference performance through perturbation. **(a)** Schematic of three types of perturbation to the E-I balanced circuit, indicated by color. These stimulation protocols were applied through: I-only, E-only, or randomly selected cells with external input. Specifically, a 1 ms impulse is applied every 20 ms. **(b)** The I-only protocol provides an overall improvement in inference at lower network strength. The distribution is from 30 instantiations. The *W* parameter in the horizontal axis is the “ground truth” synaptic coupling strength in our LIF simulation.

Finally, detecting weak interactions remains limited by the intrinsic signal-to-noise ratio of spontaneous activity. To address this, we explore an experimental strategy inspired by the “non-equilibrium response approach” [14]: driving the system to uncover hidden structure. As shown in Fig 8, periodically injecting current into inhibitory neurons improves the inference of weak weights without significantly compromising the detection of strong ones. This suggests future directions to combine FLEC with targeted perturbations (e.g., optogenetics), offering a path to resolve the full dynamic range of neural circuits.

## Materials and methods

### FLEC protocol

We here lay out the five steps in FLEC’s inference of effective coupling and neuronal response function:

- **Converting spike trains into network state space:** Given the population spike train of *N* neurons across time, the *i*th neuron that spikes at time *t* has $s_{i}(t)=1$, otherwise 0 at all times. The temporal resolution of our numerical simulations and that of the retinal data are both 0.1 ms. We choose a larger time window (t−B,t] to slide through the time series and convert spikes into a binary network state representation: $𝐬(t)=\left(s_{1}(t),s_{2}(t),...,s_{N}(t)\right)\in \{0,1{\}}^{N}$.

The window size *B*—typically 20 ms—is chosen to be larger than the numerical timestep (0.1 ms) but small relative to the typical inter-spike interval, so that state transitions correspond to individual neurons entering or leaving the window. In this regime, the dynamics is approximately an asynchronous continuous-time jump process in which only one neuron changes state at a time. In the rare case that spikes from multiple neurons enter the window within the same simulation timestep, we assign the transition to a single neuron by randomly choosing one. This enforces a well-defined single-neuron transition required for the mapping to a continuous-time jump process.

- **Extracting empirical flux counting constraints:** Given the network state representation, we compute the total dwell time $\tau_{𝐱}=\sum_{k:\;𝐬(t)=𝐱}\left(t_{k\text{-th left}}-t_{k\text{-th enter}}\right)$ for a network (configuration) state 𝐱, which is the sum of all the dwell times. We also compute the total transition counts $C_{𝐱,𝐲}=(\text{\# of }𝐱\mapsto 𝐲)$.
- **Computing the transition rate matrix:** With the two counting statistics measured, we then calculate the transition rate matrix: $R_{𝐱,𝐲}=C_{𝐱,𝐲}/\tau_{𝐱}$. This captures the normalized tendency to transition from 𝐱 to 𝐲.
- **Inferring the effective coupling and response function:** Given transition rates in the state space, the inference follows a procedure explained in the main text. This includes Eq. (2) for firing rate, Eq. (3) for effective coupling, and Eq. (4) for the response function. Furthermore, one can conduct coarse-graining procedures derived in Appendix C in S1 File.
- **Max Cal reconstruction of spike train:** The transition rate matrix $R_{𝐱,𝐲}$ can uniquely specify a Markov jump process (continuous-time Markov chain). According to the principle of Maximum Caliber (Max Cal), that Markov chain is the minimum model given the counting statistics $\left(\tau_{𝐱},C_{𝐱,𝐲}\right)$ [17,44,45]. The Markov model can then generate spike trains and inter-spike-interval statistics for each neuron. See Appendix D in S1 File for more information about Max Cal.

### Spiking neural network simulation

We simulated leaky-integrate-and-fire (LIF) circuits using the Euler method with time step d*t* of 0.1 ms. The voltage dynamics of the *i*-th neuron follows:


$$\begin{array}{c} \tau_{\text{m}}\frac{\text{d}v_{i}}{\text{d}t}=v_{\text{r}}-v_{i}+RI_{i}(t)+\xi_{i} \end{array}$$ (6)

where *v* is reset to $v_{\text{r}}=-65$ mV if it passes the threshold $v_{\text{tr}}=-50$ mV and is marked as a spike. The only place we change these biophysical parameters is in Fig 2, where neuron 2 has $v_{\text{tr}}=-60$mV. The membrane potential time constant $\tau_{\text{m}}=10$ ms and resistance *R* = 1 are fixed for all neurons. The time-varying white noise ξ has an amplitude *D*, following $\langle \xi (t)\xi (t^{\prime })\rangle =2D\;\delta (t-t^{\prime })$. The (total) synaptic current $I_{i}$ received by neuron *i* follows:


$$\begin{array}{c} \frac{\text{d}I_{i}}{\text{d}t}=-\frac{I_{i}}{\tau_{\text{s}}}+\Sigma_{j}W_{ji}\Sigma_{t_{jk}<t}\delta (t-t_{jk}) \end{array}$$ (7)

where $\tau_{\text{s}}=5$ ms is the synaptic time constant, $W_{ji}$ is the synaptic weight from neuron *j* to neuron *i*, and the *k*th spike from neuron *j* at time *t* is denoted $t_{jk}$. In these motifs, we use unit excitatory streng*t*h and report weights scaled by *W*. We simulated ~100 seconds of spikes, then embedded the network spike train in the network states.

For the large spiking network in Fig 6, we use Brian 2 for spike train simulation [46]. We use a random network with 80 excitatory neurons and 20 inhibitory neurons with sparse connectivity (connection probability *p* = 0.2). Single neuron spiking properties are the same as small circuits. The synaptic strengths are chosen so the network generates stationary irregular spiking patterns [47].

### GC and GLM inference

We used packages statsmodels [48] to perform Granger causality (GC) analysis and pyglmnet [49] to fit Generalized linear models (GLM).

GC is a common tool to infer connectivity from activity patterns in neuroscience [9,12,32]. The effective coupling is determined through fitted weights in an autoregressive process. To better fit GC with continuous data, we used the raw voltage of the simulated neurons to compute GC and took the mean across the regression coefficients as the effective coupling weights between neurons. The underlying model of GC analysis follows: $x_{i}(t)=\sum_{j=1}^{N}\sum_{k=1}^{p}A_{k}(i,j)x_{j}(t-k)+\varepsilon_{i}(t)$, where $x_{i}(t)$ is the voltage of the neuron *i* at time *t*. The voltage is predicted by interaction coefficients *A* from *p* steps back in time and Gaussian noise ε. By comparing s*t*atistical tests between the fit with and without interaction with other neurons, GC returns a p-value for the significance of each interaction. We extracted weights if the best p-value is below 0.05 and averaged the regression weights across delay time steps (lag time is set as 10 steps) as a proxy for effective coupling. Altering these parameter choices by a factor of two did not change the results significantly.

Similarly, we explored a modified GC approach for point processes, which we call spiking GC (spkGC) [33]. Rather than modeling continuous voltage traces with a Gaussian autoregressive process, spkGC models the binary spike train of each neuron using a logistic regression model with spike-history terms: $P\left(s_{i}(t)=1\right)=\sigma \left(\beta_{0,i}+\sum_{j=1}^{N}\sum_{k=1}^{L}\beta_{ij,k}s_{j}(t-k)\right)$, where s(t)∈{0,1} denotes whether neuron *i* spikes at time *t*. We include *L* = 10 previous time bins, as in standard GC, and σ(·) is the logistic function. The model was fit using logistic regression wi*t*h a small ridge regularization term for numerical stability. The signed effective coupling from neuron j to neuron i was estimated by summing the fitted history coefficients.

GLM has been widely applied for modeling neural coupling and stimulus encoding [2,6,10,11,14,31]. The effective coupling is determined through the linear weights before it passes through a spiking nonlinear function. We built design matrices for the population voltage traces lagged in time. The observations are binary spike trains, so we chose to model them with binomial GLM. The spike train of neuron *i* at time *t* follows $y_{i}(t)\sim \mathrm{Bernoulli}\left(\sigma \left(\beta_{0,i}+\sum_{j=1}^{N}\sum_{k=1}^{P}\beta_{i,j,k}x_{j}(t-k)\right)\right)$. The nonlinear function σ is logistic, the baseline coefficient is $\beta_{0}$, and the effect of the neuron *j* at *k* steps back is $\beta_{i,j,k}$. Specifically, we chose the lag time as 15 and fit without any elastic-net penalty. The effective couplings are inferred through the averaged coefficients.

## Supporting information

S1 FileAppendices A–D and Figs A–I. Appendix A: Robustness to System Parameters. Appendix B: Robustness to Sampling Parameters. Appendix C: Relation between Marginal and Conditional Couplings. Appendix D: Maximum Caliber infers Markov Jump Process as the minimal model.**Fig A.** FLEC is robust under different perturbations across circuits. (a) The cosine angle between inferred and true weights as a function of the strength of network weights. (b–d) Same as (a), but for different noise levels, synaptic delay, and differences in synaptic weights, respectively. Solid lines show the mean and shaded regions show the standard deviation from 10 instantiations. **Fig B. Spike train reconstruction is optimal at intermediate noise level across network motifs.** The KL divergence between the ISI of the LIF simulation and Max Cal reconstruction as a function of noise strength, *D*, is shown in black. The cosine angle between LIF network weights and inferred $w_{ij}$ is shown in red. The same analysis is performed for four different network motifs, with schematics of the connectivity shown in the panels. **Fig C. Inference performance across combinations of biophysical parameters.** The cosine angle (c.a.) between inferred weights and the true synaptic connections in an E-I balanced motif with LIF neurons. Parameters are scanned across different network strengths *W* and noise amplitudes *D*. **Fig D. Window size perturbative effects on inference.** The inter-spike interval across three neurons in an E-I balanced motif is shown in the middle curve. For the same motif, we compute states using five different window sizes *B* and infer the coupling weights $w_{ij}$. The sign and trend of the inferred weights are robust within a range of window sizes. **Fig E. Example motifs in the retinal spike train.** (a) Inferred coupling (top) and coarse-grained (bottom) analysis for a selected triplet of retinal ganglion cells. (b) is the example shown in main Fig 7, whereas (a) and (c) are triplets with high and low average firing rates. Triplets (a), (b), and (c) have on average 7844, 3385, and 160 observed spikes, respectively. Note that at low firing rate, the left three weights cannot be inferred due to the lack of observations of state transitions. **Fig F. Time window effect on coupling inferred from retinal spike trains.** (a) Inferred coupling (top) and coarse-grained (bottom) analysis for a selected triplet of retinal ganglion cells. The sliding window size is *B* = 20 ms. (b) Same triplet of spike trains analyzed with *B* = 150 ms window size. **Fig G. Effects of finite data size.** Cosine angle between inferred and true weights as a function of the length of the simulated spike trains. The minimum number of state transition counts for inferring $w_{j,i}$ in the simulation is shown on the right y-axis. Simulation is conducted with the same E-I circuit as in Fig 2. Error bars indicate standard deviation across 10 instantiations. **Fig H. Sampling different neurons for the three-neuron motif from retinal spike trains.** (a) The inferred weights $w_{ij}$ of six example couplings shown in Fig 7B. Neurons 1, 2, and 3 have the same IDs as in Fig 7, but here each panel fixes a pair from these three neurons and samples the remaining neuron across the dataset. The sampled neuron is rank ordered by its average firing rate across the full recording. (b) Same as (a), but showing comparison between the driven coupling $w_{ij}^{\prime }$ and the conditioned coupling $w_{ij}$. **Fig I. Learning curve with Max Cal inference.** (a) Inference for a continuous-time Markov chain (MJP) across accumulation of different groups of observables on the x-axis. The optimized KL term (top) and cosine angle between inferred and ground-truth weights (bottom) are shown. (b) Same as (a), but for a finite spike train simulated from a motif of three leaky-integrate-and-fire (LIF) neurons.(PDF)
