# Supplementary material for: Inferring effective neuronal circuits via network flux counting
Source: PLoS Comput Biol. 2026 Sep 15;22(9):e1014763. doi: 10.1371/journal.pcbi.1014763 (PMC13592720; doi:10.1371/journal.pcbi.1014763)
Supplement: Supplementary file 1 [file pcbi.1014763.s001.pdf]

Supporting Information

Appendix A: Robustness to System Parameters

Here, we test the robustness of FLEC inference performance across different types of parameter variations. The analysis is done across three types of motifs to explore how inference depends on the ground truth motifs. The variation includes:

1. **Weights**: the synaptic weights of the circuit (W_{ji}) that have unitary excitatory weights are rescaled by a positive scalar W.

2. **Noise**: the noise strength D that controls the magnitude of the time-varying white noise is altered. The combination of weight and noise is controlling the overall signal-to-noise ratio of the inference problem.

3. **Delay**: a synaptic connection that is common to all motifs (neuron 1 $\rightarrow$ neuron 3) is selected, then the transmitted spike timing through this connection is delayed by a discrete number of $\mathrm{d}t$ time steps.

4. **Difference**: the synaptic connection common to all motifs is rescaled by a positive scalar, while keeping the other connection strengths fixed. This creates a form of heterogeneous connectivity.

As a result, we find that the trend of inference alters monotonically upon different types of perturbation, and this trend roughly holds across all types of perturbations. Intuitively, the inference of FLEC degrades with small weight and large noise, which corresponds to lower signal-to-noise for information about the structure given activity. Synaptic delay has little effect in E-I balanced and common-input circuits. This robustness comes from two mechanisms. If the delay difference between target neurons exceeds the sliding window, their spikes do not overlap. The delayed response simply adds to the spontaneous firing rate (f_i) without altering the pairwise coupling (w_{ji}). If the delay difference is shorter than the window, the target neurons fire while the common driver (k) remains active. Because FLEC computes w_{ji} only from states where

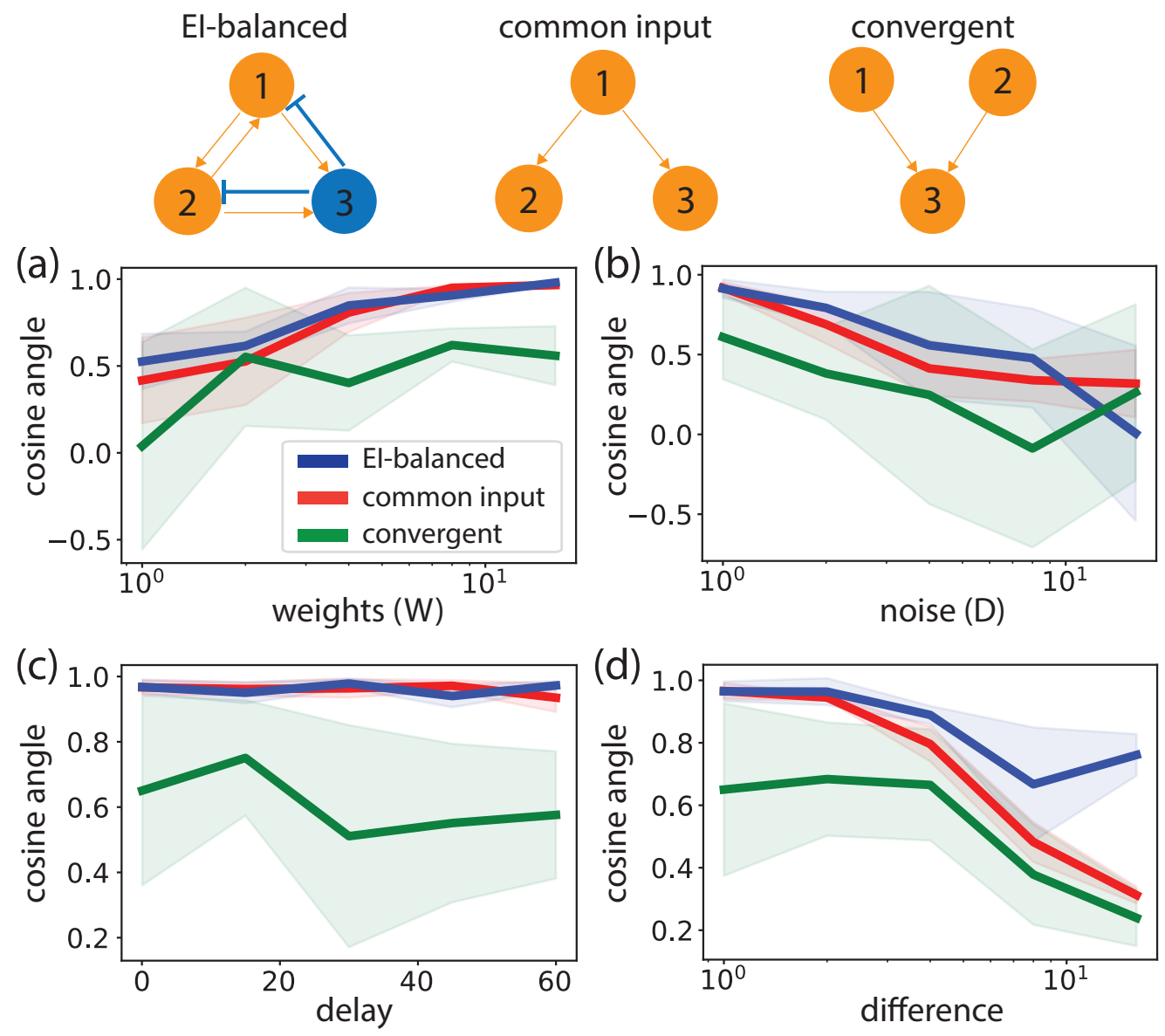

Fig A. FLEC is robust under different perturbation across circuits. (a) The cosine angle between inferred and true weights as a function of the strength of network weights. **(b-d)** are the same as (a) but for different noise levels, synaptic delay, and difference of synaptic weights, respectively. Solid lines show mean and shadows show std from 10 instantiations.

the common driver is silent ($k = 0$), it structurally excludes the correlated common input. Lastly, the difference in connection strength also leads to poorer performance. As expected, successful network inference is related to the signal-to-noise ratio in the measured time series and the differentiability of the network parameters. However, this systematic analysis shows that the working range for inference is rather wide.

In addition to the inferred connectivity, we explore how the inference performance of the inter-spike interval (ISI) distribution from Max Cal minimal model is related to that of the circuit parameter. Specifically, we focus on the dependence on noise strength D. Across different circuit motifs, we find that there is a regime of optimal noise strength that provides good circuit inference as well as the ISI reconstruction. We reason that this parameter regime produces spikes that are more "Poissonian", equivalent to a Markov jump process, and thereby providing better circuit inference.

The structural signal is controlled by a scalar W, which scales the network matrix W_{ij}, whose original excitatory strength is one. The noise amplitude $\langle \xi(t)\xi(t') \rangle = 2D\delta(t - t')$. We scan through a range of W and D and record the inference performance for LIF network simulations. In agreement with the signal-to-noise ratio, the correlation between inferred weights and true synaptic connections is large at strong

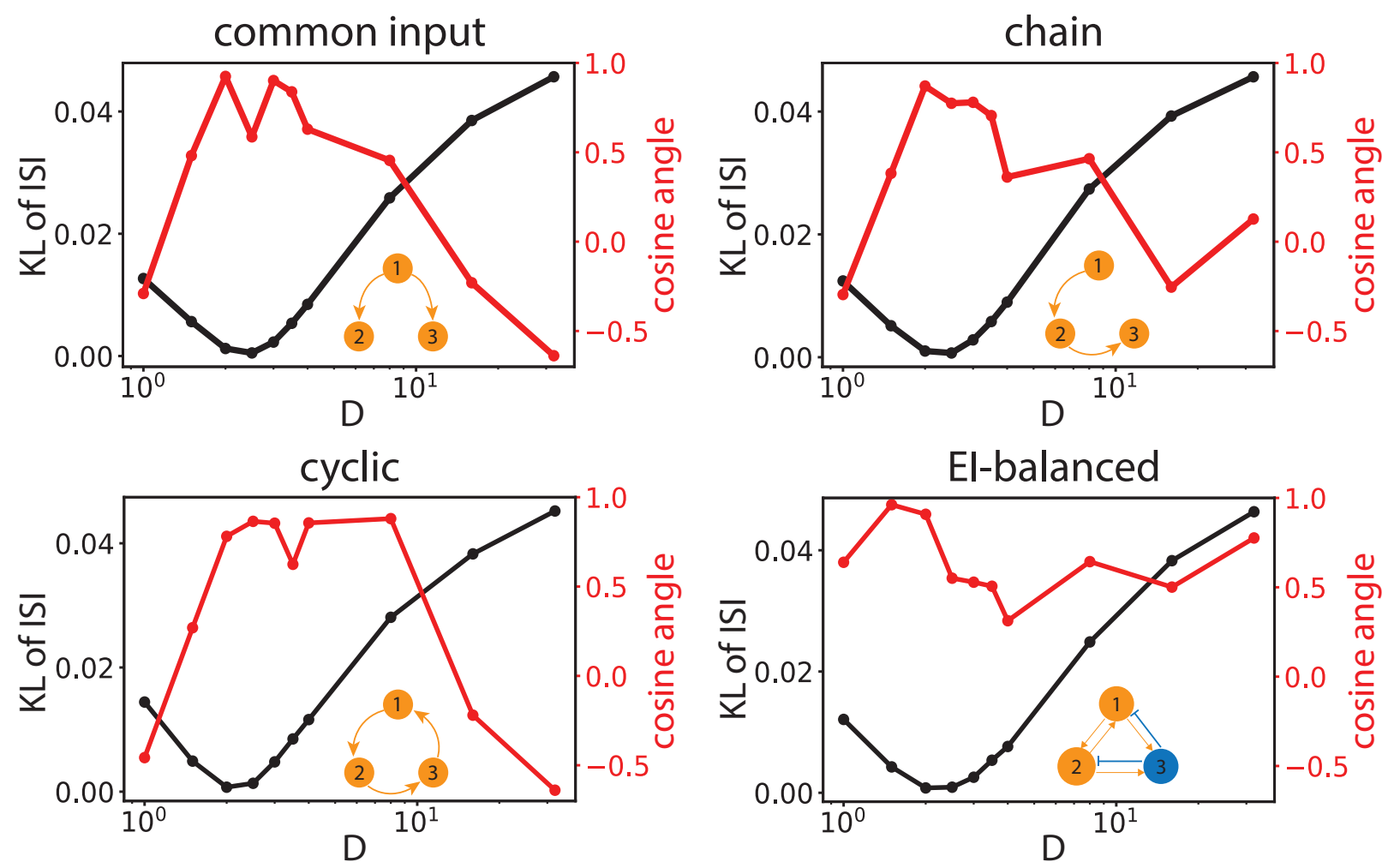

Fig B. Spike train reconstruction is optimal at intermediate noise level across network motifs. The KL divergence between ISI of LIF simulation and Max Cal reconstruction as a function of noise strength, D, is shown in black. The cosine angle between LIF network weights and inferred w_{ij} is shown in red. The same analysis is done for four different network motifs, with schematic of the connectivity shown in the panels.

network strength and weak noise strength (Fig C). Unless otherwise mentioned, we used $D = 2$ and $W = 20$ throughout the LIF simulations. Since the weights are on the same scale as the gap between resting potential and spiking threshold (15 mV), this is a high signal-to-noise ratio parameter setting we explored in Figs 2-5.

Appendix B: Robustness to Sampling Parameters

Effects of Window Size The FLEC inference procedure is non-parametric, with only one data-processing parameter that is the sliding window size to convert spike train

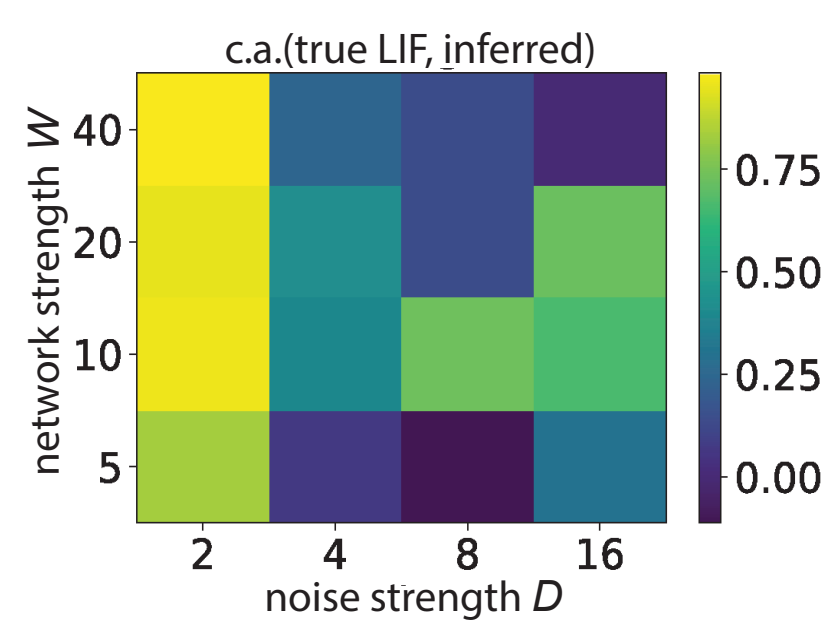

Fig C. Inference performance across combinations of biophysical parameters. The cosine angle (c.a.) between inferred weights and the true synaptic connection in an E-I balanced motif with LIF neurons. Parameters are scanned across different network strengths W and noise amplitudes D.

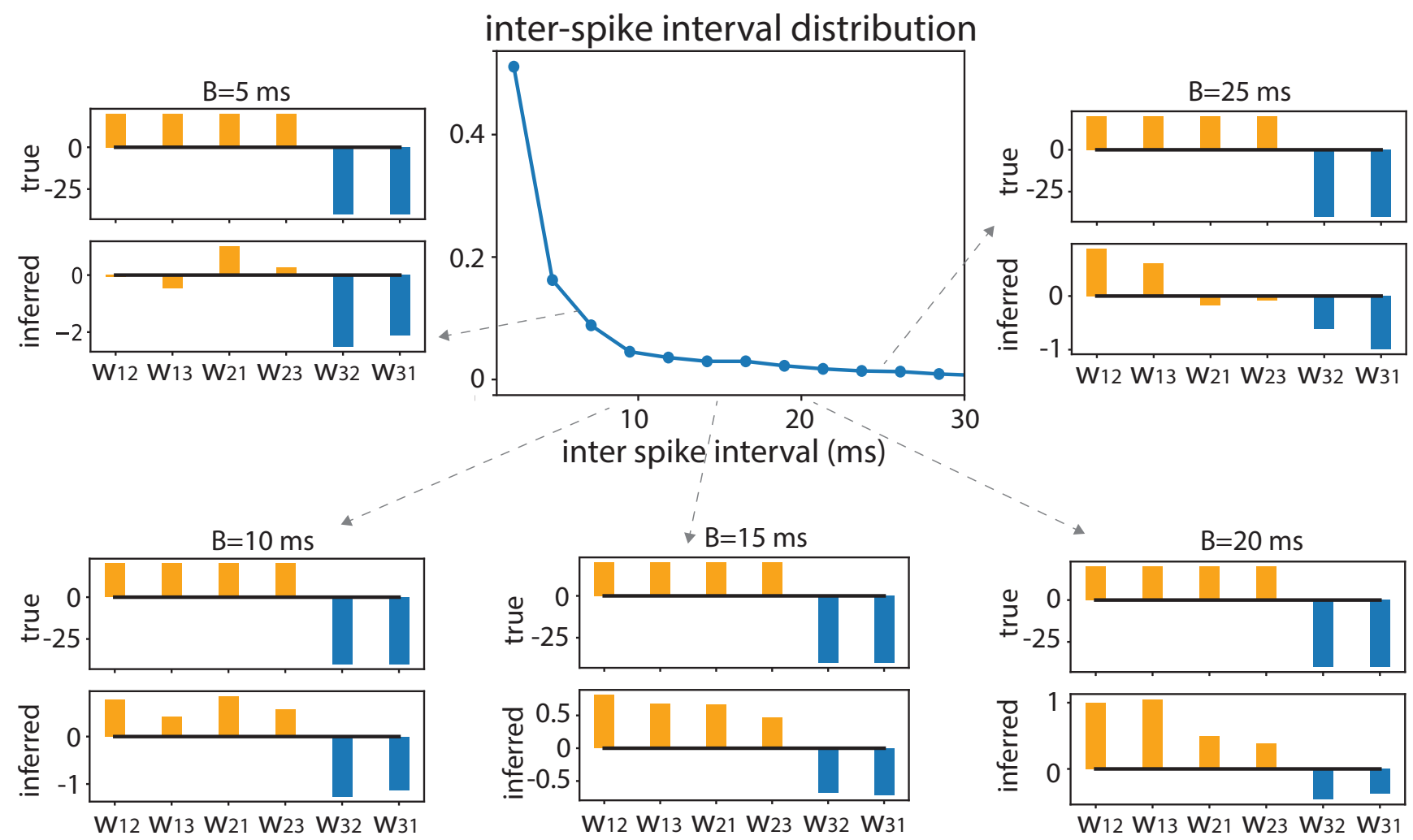

Fig D. Window size perturbative effects on inference. The inter-spike interval across three neurons in a E-I balanced motif is shown in the middle curve. For the same motif, we compute states using five different window sizes B and infer the coupling weights w_{ij}. The sign and trend of the inferred weights are robust within a window range.

data into transitions in the neural state space. The size of this sliding window can affect transition statistics. To seek a consistent choice of this window size, we analyze the inter-spike interval (ISI) distribution from all neurons in an LIF network with E-I balanced motif. We explore potential correlation between the inference performance of different window sizes and the ISI distribution—hoping to find rules of thumb to select the window size from ISI distribution in future applications.

At a small window size close to the mass of short ISI events, the inferred weights have more negative values. This is due to many observed single neuron states that transition back to full-silence and the lack of richer network states when the bin size is close to the refractory period. In contrast, at much larger time window, the events might be lumped together and lose information about the circuit. Empirically, the inferred weights are more robust within a range of intermediate time window size (Fig D).

Window size effect on inferring retinal coupling We demonstrated an example retinal neural motif with negligible excitation in Fig 7B. Empirically, most of the triplet motifs from the retinal network we selected show negative coupling or only weak excitation (Fig E, Fig F(a)). This seems consistent with known physiology of the retinal ganglion cells (RGC)—there are unlikely to be direct synaptic (monosynaptic)

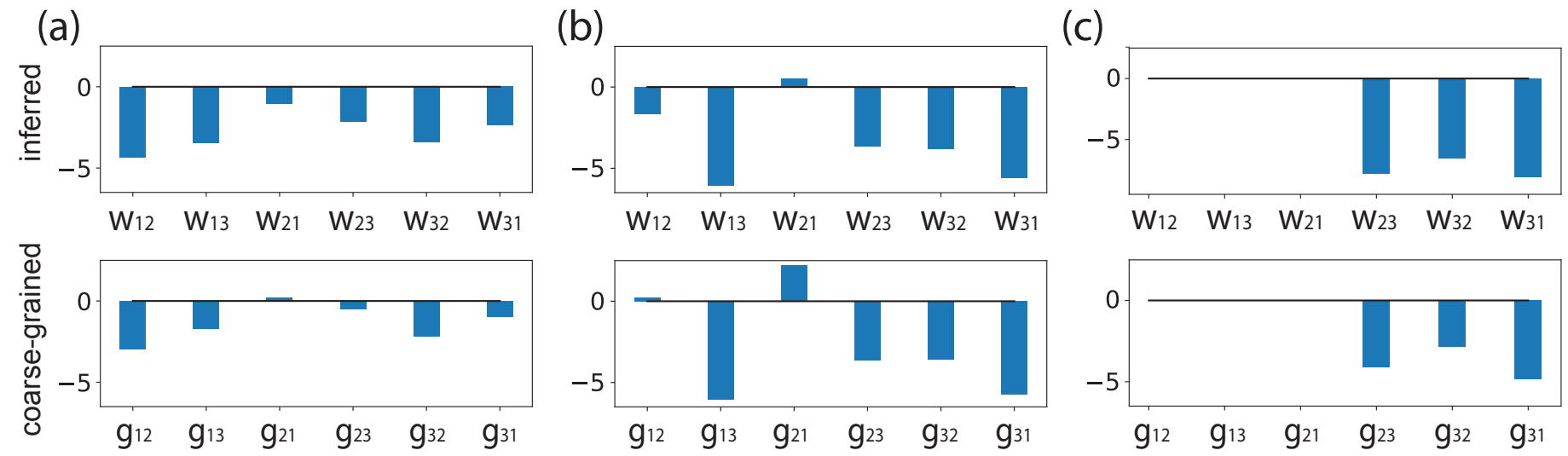

Fig E. Example motifs in the retinal spike train. (a) Inferred coupling (top) and coarse-grained (bottom) analysis for a selected triplet of retinal ganglion cells. (b) is the example shown in the main Fig 7, whereas (a) and (c) are triplets with high and low average firing rates. (a),(b),(c) triplets have on average 7844, 3385, and 160 observed spikes, respectively. Note that at low firing rate, the left three weights cannot be inferred due to the lack of observations of state transitions.

connections between RGCs to lead to direct mutual excitation. As a functioning motif, many RGCs have lateral inhibition between each other [1–3]. The inference reveals consistent negative weights at the 20 ms timescale, which is short enough for direct synaptic coupling.

We verify that the state transition counts in the retina data are comparable to those of the simulated spike train sufficient for network inference (order of ∼1000 transitions). Additionally, we confirm that perturbing the time window size for the retinal spike trains has a similar effect on weight inference as the simulated spikes (Figs D and Fig F). These results indicate that consistent conclusions can be drawn from finite but sufficient measurements.

The retinal spike train conveys information about the complex visual stimuli, in this case a moving bar following a random walk [4]. As indicated in the paper [4], the stimulus has correlation on the timescale of $100-200$ms and moves continuously across the receptive field of the measured RGCs. We seek to characterize such "functional coupling" induced by spatiotemporally correlated external input by re-analyzing the spike train with a larger window (Fig F(b)). As a result, we find that at larger window size $B = 150$ ms, the inference shows functional coupling with more structure— i.e. more excitatory coupling. This seems to reflect the long time-scale signal introduced through stimulus or more complex retinal computation, which can be studied through more detailed encoding models in the future [5–7].

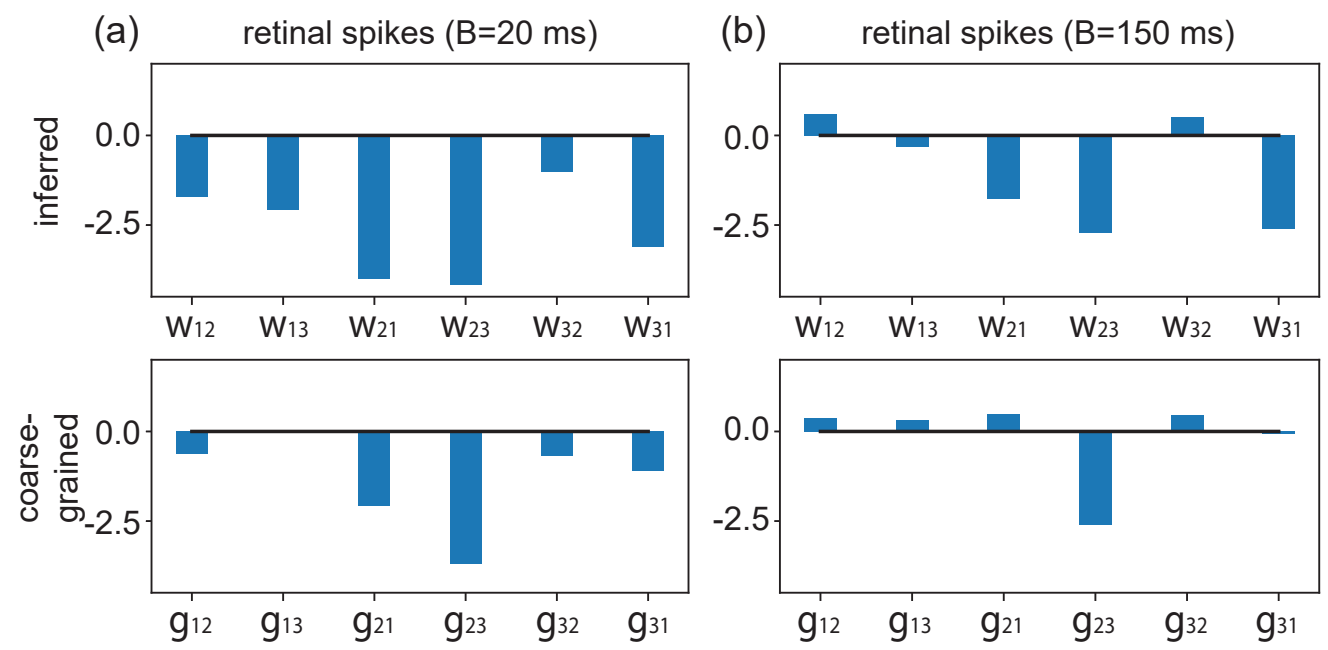

Fig F. Time window effect on coupling inferred from retinal spike trains. (a) Inferred coupling (top) and coarse-grained (bottom) analysis for a selected triplet of retinal ganglion cells. The sliding window size is $B = 20$ ms. **(b)** Same triplet of spike trains analyzed with $B = 150$ ms window size.

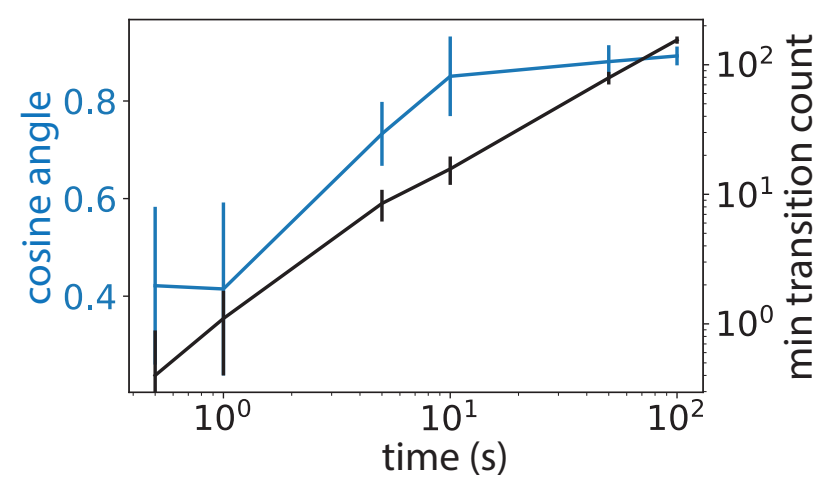

Fig G. Effects of finite data size. Cosine angle between inferred and true weights as a function of the length of the simulated spike trains. The minimum number of state transition counts for inferring $w_{j,i}$ in the simulation is shown on the right y-axis. Simulation is conducted with the same EI circuit in Fig 2. Error bars indicate standard deviation across 10 instantiations.

Finite Sample Effects For LIF spike trains, we study how inference performance scales with the length of spike trains (Fig G). In an E-I balanced circuit, performance improves with the length of data and saturates after it is approximately longer than ~ 10s. We further quantify the corresponding transition statistics and found empirically that reliable network inference requires observing on the order of ~ 100 counts of the least frequent transitions (Fig G). This empirical scaling relation provides a practical guideline for evaluating FLEC performance under finite data conditions.

Appendix C: Relation between Marginal and Conditional Couplings

While we demonstrate inference from statistics for the full neural state space in the main text, one can coarse grain dynamics in this framework through calculating the marginal statistics. For instance, within each triplet circuit motif, we can take any two of the

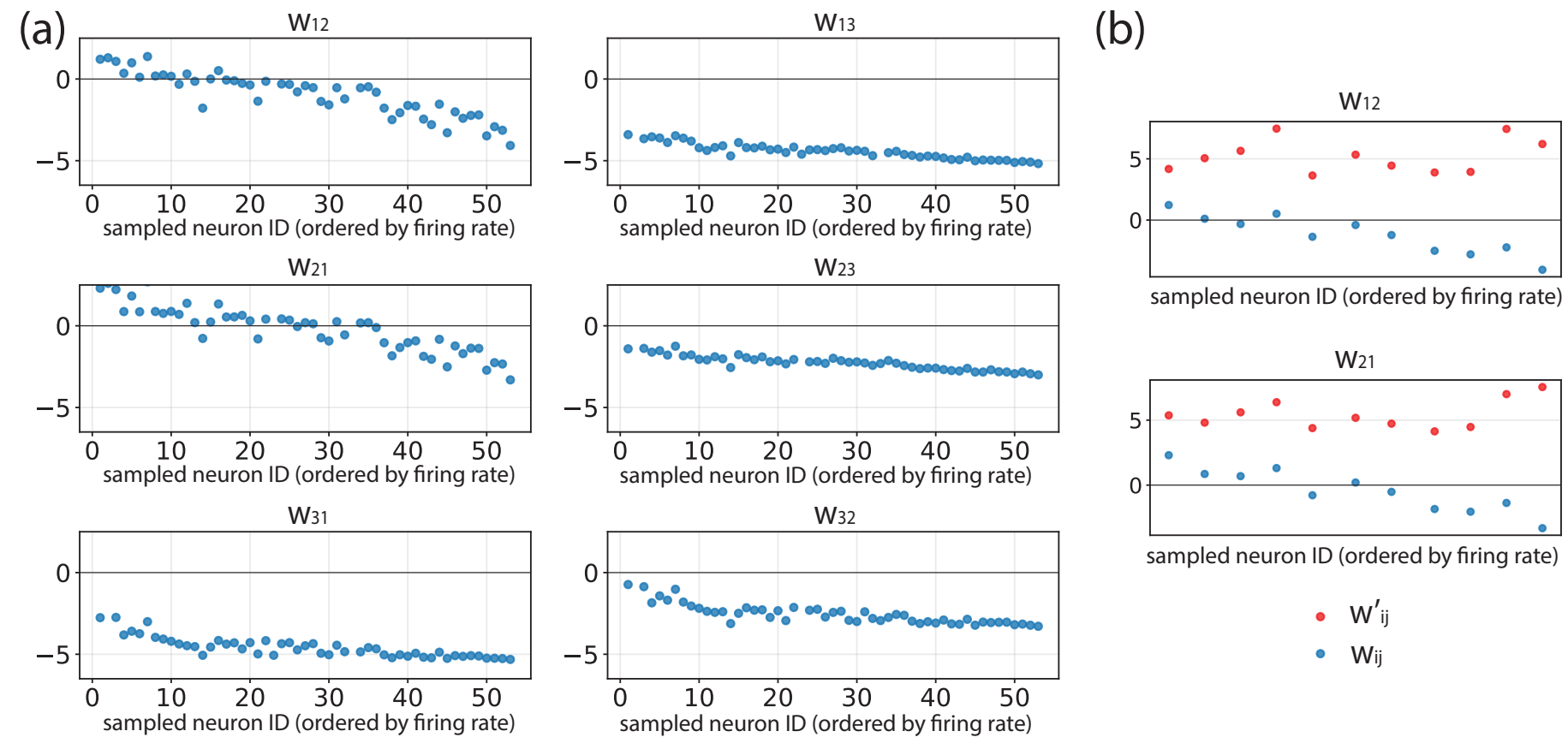

Fig H. Sampling different neurons for the three-neuron motif from retinal spike trains. (a) The inferred weights w_{ij} of six example couplings shown in Fig 7B. The neurons 1,2,3 are the same ID as the Fig 7, but here each panel fixes a pair from these three neurons and samples the remaining neuron across the data set. The sampled neuron is rank ordered by its average firing rate across the full recording. **(b)** Same as (a) but showing comparison between the driven coupling w' and the conditioned coupling w.

three neurons and repeat the same procedure to infer a marginalized effective coupling:

$$g_{ij} = \ln \frac{R_{(10),(11)}}{R_{(00),(01)}}. \tag{1}$$

This focuses only on paired interactions, but can provide consistent information about synaptic connections. This is consistent with past work demonstrating the statistical power of pairwise interactions in the neural network [5, 8, 9]. It can be shown that the coarse-grained coupling g_{ij} is actually the conditional average among the triplet effective coupling $w_{i,j}$ and $w_{ik,j}$:

$$g_{ij} = \ln \frac{[\text{flux } (10^*) \mapsto (11^*)]/[\text{time in } (10^*)]}{[\text{flux } (00^*) \mapsto (01^*)]/[\text{time in } (00^*)]} \tag{2a}$$

$$= \ln \frac{[\tau_{(\ldots 100 \ldots)} e^{w_{i,j}} + \tau_{(\ldots 101 \ldots)} e^{w_{ik,j}}]/\tau_{(\ldots 10* \ldots)}}{[\tau_{(\ldots 000 \ldots)} + \tau_{(\ldots 001 \ldots)} e^{w_{k,j}}]/\tau_{(\ldots 00* \ldots)}} \tag{2b}$$

where $\tau_{(\ldots X \ldots)}$ represents the total time when pattern X occurs in the network (e.g. $X = 10*$ is when neuron j is silent whereas i is firing and k can be either state, and $X = 010$ is further conditioning on k being silent). When coupling from i to j is excitatory, we expect both $w_{i,j} > 0$ and $w'_{i,j} = w_{ik,j} - w_{k,j} > 0$ because the former gives the effective coupling conditioning on the silence of neuron k whereas the latter gives that conditioning on the active state of neuron k. This, however, does *not* directly

imply $g_{ij} > 0$. This highlights the complexity of coupling inference. Below we discuss how g, w, w' relate to each other in idealized limits.

Equation (2b) demonstrates that the marginal coupling g_{ij} is not a simple average, but a state-dependent mixture of the basal coupling $w_{i,j}$ (when the third neuron is silent), the driven coupling $w'_{i,j}$ (when the third neuron is firing), and the background drive $w_{k,j}$ (from the third neuron to j). To formalize this, we define the conditional probabilities of the background neuron k being silent (given that neuron j is silent) when neuron i is active or silent, respectively:

$$p_1 = \frac{\tau_{(\dots 100\dots)}}{\tau_{(\dots 10*\dots)}}, \quad p_0 = \frac{\tau_{(\dots 000\dots)}}{\tau_{(\dots 00*\dots)}}$$

where $1 - p_1 = \tau_{(\dots 101\dots)}/\tau_{(\dots 10*\dots)}$ and $1 - p_0 = \tau_{(\dots 001\dots)}/\tau_{(\dots 00*\dots)}$. By substituting these probabilities into Eq. (2b) and expressing the joint coupling as $w_{ik,j} = w_{k,j} + w'_{i,j}$, the marginal coupling can be rewritten exactly as:

$$e^{g_{ij}} = \frac{p_1 e^{w_{i,j}} + (1 - p_1)e^{w_{k,j} + w'_{i,j}}}{p_0 + (1 - p_0)e^{w_{k,j}}}$$

This formulation analytically explains several empirical network phenomena by evaluating the system under different activity limits:

- **Low Background Activity:** If the background neuron k has a very low firing rate, it is predominantly silent, meaning $p_1 \to 1$ and $p_0 \to 1$. In this limit, the marginal coupling reduces to the basal conditional coupling:

$$e^{g_{ij}} \approx \frac{1 \cdot e^{w_{i,j}} + 0}{1 + 0} = e^{w_{i,j}} \implies g_{ij} \approx w_{i,j}$$

- **High Background Activity:** Conversely, under high global activity where k is predominantly active, the conditional probabilities approach zero ($p_1 \to 0$ and $p_0 \to 0$). The equation then simplifies to:

$$e^{g_{ij}} \approx \frac{0 + 1 \cdot e^{w_{k,j} + w'_{i,j}}}{0 + 1 \cdot e^{w_{k,j}}} = e^{w'_{i,j}} \implies g_{ij} \approx w'_{i,j}$$

  In this highly active regime, the marginal coupling g_{ij} effectively reflects the driven coupling $w'_{i,j}$.

- **Strong Correlation:** If neurons i and k are highly correlated, neuron k will likely fire when i fires ($p_1 \to 0$) and remain silent when i is silent ($p_0 \to 1$). Taking this limit yields:

$$e^{g_{ij}} \approx \frac{0 + 1 \cdot e^{w_{k,j}+w'_{i,j}}}{1+0} = e^{w_{k,j}+w'_{i,j}} \implies g_{ij} \approx w'_{i,j} + w_{k,j}$$

  In this highly correlated regime, the sign and magnitude of the marginal coupling g_{ij} are modulated by $w_{k,j}$ (the direct influence of k on j).

Consequently, differences between the marginal g_{ij} and the conditional $w_{i,j}$ do not simply flag finite-data errors or spurious links; rather, they quantitatively capture the extent to which unobserved or marginalized network activity (such as shared sensory excitation or lateral inhibition) modulates the local pairwise interactions.

Appendix D: Maximum Caliber infers Markov Jump Process as the minimal model

The principle of Maximum Entropy (Max Ent), originating from statistical physics, is a general framework for inferring the least-biased static probability distribution given a set of constraints [10–14], which has been proven useful in neuroscience [8, 9, 15–17]. The principle of *Maximum Caliber* (Max Cal) extends Max Ent to infer dynamical processes from constraints on paths or trajectories [18–20]. When the available information consists of state distributions and first-order transition counts (fluxes), Max Cal infers a Markov process (continuous-time **Markov Jump Process (MJP)** in the discrete-state, continuous-time case) as the minimal model [21–23]. This inference comprises two distinct tasks: (1) establishing that the model form is *Markovian*, and (2) inferring the specific *transition rate parameters* from observation. In the main text, we rely on the first task to justify our use of an MJP model. We then bypass the second task by empirically measuring the *complete set* of fluxes from sufficient data, which directly provides the transition rates.

When one must infer the rates from an incomplete set of constraints (Task 2), the problem is equivalent to finding the MJP that satisfies the constraints while being as close as possible to a prior MJP model. This is achieved by minimizing the

Kullback-Leibler (KL) divergence rate between the posterior process with transition rates R and a prior process R^0. The optimization problem is to find the posterior transition rates $R^*_{\xi,\eta}$ that minimize this rate, $d(R||R^0)$ (See the Supplemental Information of [24] and the references within):

$$R^*_{\xi,\eta} = \arg\min_{R \in \mathcal{R}_c} d(R||R^0) \tag{3}$$

where the KL rate is given by:

$$d(R||R^0) = \sum_{\xi \neq \eta} \pi_\xi R_{\xi,\eta} \ln \frac{R_{\xi,\eta}}{R^0_{\xi,\eta}} - \pi_\xi R_{\xi,\eta} + \pi_\xi R^0_{\xi,\eta}, \tag{4}$$

$\pi_\xi(R)$ is the steady-state distribution of the process with rates $R_{\xi,\eta}$, and $\mathcal{R}_c$ represents all possible MJP models that satisfy the given set of constraints c.

Fig I shows the results of performing this Task-2 inference procedure. Network inference as experimental constraints are progressively incorporated into the MaxCal optimization (listed on the horizontal axis). We increment the set of constraints from including only the time fractions of different state τ, to including fluxes that can infer $f_i, w_{ij}, w_{ij,k}$, etc. As more information is incorporated, the posterior KL rate d^* increases monotonically, signifying a gain in information and a more accurate model (top panel). Crucially, the cosine angle between the inferred and true weights—our measure of inference accuracy—improves and saturates quickly once the key flux information (f_i, w_{ij}) is included (bottom panel). This finding illustrates that the core information about the effective couplings is contained within a specific, measurable set of transition fluxes, reinforcing that with sufficient data, the complex optimization of Task 2 can be bypassed.

In computing Max Cal in Fig I, the Max Cal objective in Eq. (3) was optimized with Sequential Least Squares Programming (SLSQP) in Python. The equality constraint imposed by minimizing the squared error between model and observables and the scalar objective is the KL term. We use a uniform stationary prior for K^0_{ij}. We empirically verify that the tolerance and initial conditions have negligible effects on the convergence. Upon convergence, the equality constraints are down to the order of 10^{-12}.

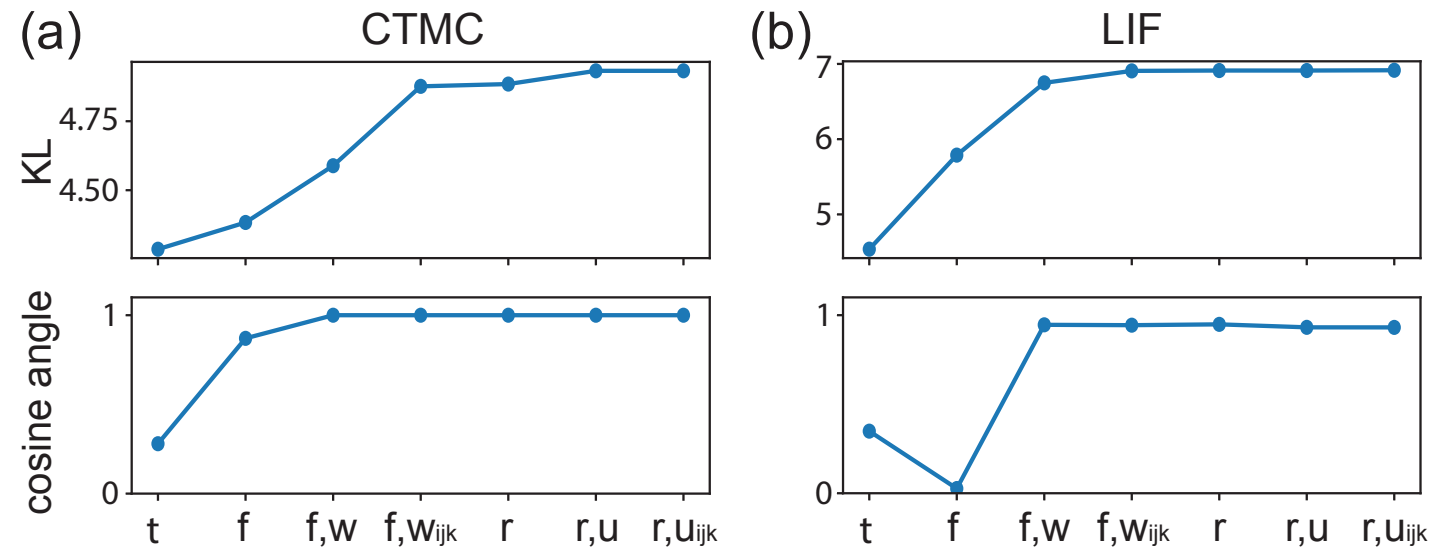

Fig I. Learning curve with Max Cal inference. (a) Inference for continuous-time Markov chain (MJP) across accumulation of different groups of observables on the x-axis. The optimized KL term (top), cosine angle between inferred and ground-truth weights (bottom). **(b)** Same as (a) but for a finite spike train simulated from a motif of three leaky-integrate-and-fire (LIF) neurons.

References

1. Luo L. Architectures of neuronal circuits. Science. 2021;373(6559):eabg7285.
2. Rieke F, Warland D, Van Steveninck RdR, Bialek W. Spikes: exploring the neural code; 1999.
3. Masland RH. The fundamental plan of the retina. Nature neuroscience. 2001;4(9):877-86.
4. Palmer SE, Marre O, Berry MJ, Bialek W. Predictive information in a sensory population. Proceedings of the National Academy of Sciences. 2015;112(22):6908-13.
5. Pillow JW, Shlens J, Paninski L, Sher A, Litke AM, Chichilnisky E, et al. Spatio-temporal correlations and visual signalling in a complete neuronal population. Nature. 2008;454(7207):995-9.
6. Gerstner W, Kistler WM, Naud R, Paninski L. Neuronal Dynamics: From Single Neurons to Networks and Models of Cognition. 1st ed. Cambridge University Press; 2014.
7. Granot-Atedgi E, Tkačik G, Segev R, Schneidman E. Stimulus-dependent maximum entropy models of neural population codes. PLoS computational biology. 2013;9(3):e1002922.
8. Schneidman E, Berry MJ, Segev R, Bialek W. Weak pairwise correlations imply strongly correlated network states in a neural population. Nature. 2006

Apr;440(7087):1007-12. Available from: http://www.nature.com/nature/journal/v440/n7087/full/nature04701.html. doi:10.1038/nature04701.

9. Roudi Y, Aurell E, Hertz JA. Statistical physics of pairwise probability models. Frontiers in computational neuroscience. 2009;3:652.

10. Jaynes ET. Information Theory and Statistical Mechanics. Phys Rev. 1957 May;106(4):620-30. Available from: https://link.aps.org/doi/10.1103/PhysRev.106.620. doi:10.1103/PhysRev.106.620.

11. Shore J, Johnson R. Axiomatic derivation of the principle of maximum entropy and the principle of minimum cross-entropy. IEEE Trans Inf Theor. 1980 Jan;26(1):26-37. Available from: https://doi.org/10.1109/TIT.1980.1056144. doi:10.1109/TIT.1980.1056144.

12. van Campenhout J, Cover T. Maximum entropy and conditional probability. IEEE Trans Inform Theory. 1981 Jul;27(4):483-9. doi:10.1109/TIT.1981.1056374.

13. Jizba P, Korbel J. Maximum Entropy Principle in Statistical Inference: Case for Non-Shannonian Entropies. Phys Rev Lett. 2019 Mar;122(12):120601. Available from: https://link.aps.org/doi/10.1103/PhysRevLett.122.120601. doi:10.1103/PhysRevLett.122.120601.

14. Caticha A. Entropy, Information, and the Updating of Probabilities. Entropy. 2021 Jul;23(7):895. Available from: https://www.mdpi.com/1099-4300/23/7/895. doi:10.3390/e23070895.

15. Das A, Fiete IR. Systematic errors in connectivity inferred from activity in strongly recurrent networks. Nature Neuroscience. 2020;23(10):1286-96.

16. Tkačik G, Marre O, Amodei D, Schneidman E, Bialek W, Berry MJ. Searching for collective behavior in a large network of sensory neurons. PLoS computational biology. 2014;10(1):e1003408.

17. Vasquez JC, Marre O, Palacios AG, Berry II MJ, Cessac B. Gibbs distribution analysis of temporal correlations structure in retina ganglion cells. Journal of Physiology-Paris. 2012;106(3-4):120-7.

18. Jaynes ET. The Minimum Entropy Production Principle. Annual Review of Physical Chemistry. 1980;31(1):579-601. Available from: https://doi.org/10.1146/annurev.pc.31.100180.003051. doi:10.1146/annurev.pc.31.100180.003051.

19. Pressé S, Ghosh K, Lee J, Dill KA. Principles of maximum entropy and maximum caliber in statistical physics. Rev Mod Phys. 2013 Jul;85(3):1115-41. Available from: https://link.aps.org/doi/10.1103/RevModPhys.85.1115. doi:10.1103/RevModPhys.85.1115.

20. Pachter JA, Yang YJ, Dill KA. Entropy, irreversibility and inference at the foundations of statistical physics. Nat Rev Phys. 2024 May:1-12. Publisher: Nature Publishing Group. Available from: https://www.nature.com/articles/s42254-024-00720-5. doi:10.1038/s42254-024-00720-5.

21. Csiszar I, Cover T, Byoung-Seon Choi. Conditional limit theorems under Markov conditioning. IEEE Trans Inform Theory. 1987 Nov;33(6):788-801. Available from: http://ieeexplore.ieee.org/document/1057385/. doi:10.1109/TIT.1987.1057385.

22. Lee J, Pressé S. A derivation of the master equation from path entropy maximization. The Journal of Chemical Physics. 2012 Aug;137(7):074103. Available from: https://doi.org/10.1063/1.4743955. doi:10.1063/1.4743955.

23. Ge H, Pressé S, Ghosh K, Dill KA. Markov processes follow from the principle of maximum caliber. The Journal of Chemical Physics. 2012 Feb;136(6):064108. Available from: http://aip.scitation.org/doi/10.1063/1.3681941. doi:10.1063/1.3681941.

24. Yang YJ, Dill KA. A principled basis for nonequilibrium network flows. Nat Commun. 2026 Apr;17:4980. Available from:

https://www.nature.com/articles/s41467-026-71239-9.
doi:10.1038/s41467-026-71239-9.
