# Supplementary material for: Inferring effective neuronal circuits via network flux counting
Source: PLoS Comput Biol. 2026 Sep 15;22(9):e1014763. doi: 10.1371/journal.pcbi.1014763 (PMC13592720; doi:10.1371/journal.pcbi.1014763)
Supplement: Supplementary file 2 [file pcbi.1014763.s003.pdf]

Reviewer #1: Chen and Yang introduce a novel approach to estimate the connectivity of a neural circuit from spiking activity. They treat each network state (e.g. [0,1,0]) as a node on a hypercube, with edges between those differing by a single change (e.g. compared with the previous example [0,0,0] or [1,1,0]). The rate at which those edges are traversed can be used to estimate the spontaneous rates of units, their effective coupling, and their nonlinear response functions.

The results are presented clearly with straightforward examples highlighting the advantages of this new approach. I don't have any major theoretical criticisms of the work but do have some concerns regarding the scalability of this approach to large scale in vivo datasets and a potential degenerate situation that may yield false connections.

We thank the referee for their positive evaluation. We have addressed all concerns raised below.

Major concerns:

1. While the authors raise the issue that this approach has poor scaling with the number of nodes, it is unclear how bad the problem is when going from 3 to 5 nodes, or 5 to 20, or 100. It would be informative to show the form this scaling takes, such as how the number of nodes and edges that need to be evaluated grow. In addition, it would seem as the number of units increases the sampling of specific transition rates gets lower, so how would the duration of a simulation/recording have to increase to account for this. Presumably this depends in part on the firing rate of the neurons and sliding window size of the neurons, so keep those constant to just demonstrate should be sufficient.

We thank the referee for raising this important point. We have now included a paragraph (Lines 209-217) to explicitly discuss how the number of nodes and edges scales with the number of neurons, and the corresponding empirical data requirements. In short, for an N-neuron spiking network, there are 2^N nodes and $N 2^N$ transitions. Empirically (Fig. S7), reliable inference requires at least ~100 transition counts per edge. For N=100, the number of transitions grows to 10^{32}, rendering direct global inference computationally intractable. Together, these results demonstrate that direct global FLEC inference becomes both computationally and data prohibitive for large networks and motivate the motif-based coarse-graining approach developed in the manuscript.

> *"Inspired by successful inference in small motifs, we seek to apply FLEC to larger networks. However, a scaling argument reveals the challenge of a direct application. Consider a spiking network of N neurons, each modeled by a binary state. The network has 2^N possible configurations, and under the multipartite assumption that only one change happens at an instant in continuous time, there are $N2^N$ transitions in the state space. Analytical FLEC requires counting fluxes on the $N2^N$ transitions and the dwelling times of the 2^N configurations. The dimensionality and the requirement of data grow exponentially: there are about 10^4 transitions when N=10, which grows to 10^{32} when N=100. In Fig. S7 in Appendix, we show that empirically each transition*

requires at least 100 transition counts for the inference performance to saturate. Therefore, a perturbative coarse-grained approach is needed to go beyond motifs."

2. The suggested solution for large networks of neurons is to subsample pairs or triplets. The authors show that in most cases there is a positive cosine angle between the ground truth and the subsampled triplet. However, for data collected in vivo there is no ground truth, so instead it might be helpful to know what the variance would be in the estimated effective connectivity between a pair of units. To do this, you would choose a pair and then vary across multiple runs the 3rd unit in the triplet

We thank the reviewer for this constructive suggestion. In the revised manuscript, we implemented this subsampling analysis on the retinal spike-train data. For each fixed neuron pair i,j, we repeatedly inferred the effective coupling w_ij while systematically varying the identity of the third conditioning neuron k across the recorded population. We then quantified the variability of w_ij across different choices of k. These results are shown in the new Fig. 7b and comprehensively across sampled triplets in the new Fig. S8. For example, the error bar for w_23 in Fig. 7b is obtained by fixing neurons 2 and 3 while varying the identity of neuron 1 across the recorded population. We find that the dominant inhibitory interactions are generally robust across different conditional backgrounds, whereas weaker interactions show greater variability.

3. There may be a degenerate case in this approach that would show a connection between two unconnected units. If two units both received input from the same third unit, but at slightly different lags, it would establish a fixed lagged relationship between their spiking. If the difference between the lags falls within the sliding window size, then that might show a connection between them. Would this pose a problem, and if not, can the authors show why? In cross-correlation analysis of point process data, this is usually dealt with by shuffling the data across trials (see https://protect.checkpoint.com/v2/r01/___https://pmc.ncbi.nlm.nih.gov/articles/PMC3856913/___.YzJ1OnN0b255YnJvb2s6YzpnOjg2ODUyNWJmMWZiMmYzMjI4YTViZTgxM2U3OGU3YzBhOjc6N2U4MjplMjA4ZmM0NWExOTY4YmM3ZDZhMzBkMDZlZTM4MTBmNDE4M2Q0MWI0Y2I1NjliMGU3MzJiNjgwMTc0MmUzMzc1OnQ6VDpG).

In Fig. S1(c), we have shown that FLEC is quite robust to synaptic delay in a common input motif. The delay was varied from 0 to 60 ms, from smaller to three times bigger than the window size 20 ms. The cosine angle remains high across the delay variation.

We have now added a brief discussion about this in Appendix 1. When the delay difference is shorter than the window, the target neurons fire while the common driver (k) remains active. Because the basal conditional coupling w_ji is computed only from states where the observed common driver is silent (k=0), this conditioning excludes the correlated activity attributable to that observed common input.

"As a result, we find that the trend of inference alters monotonically upon different types of perturbation, and this trend roughly holds across all types of perturbations. Intuitively,

the inference of FLEC degrades with small weight and large noise, which corresponds to lower signal-to-noise for information about the structure given activity. Synaptic delay has little effect in E-I balanced and common-input circuits. This robustness comes from two mechanisms. If the delay difference between target neurons exceeds the sliding window, their spikes do not overlap. The delayed response simply adds to the spontaneous firing rate (f_i) without altering the pairwise coupling (w_{ji}). If the delay difference is shorter than the window, the target neurons fire while the common driver (k) remains active. Because FLEC computes w_{ji} only from states where the common driver is silent (k=0), it structurally excludes the correlated common input."

Minor concerns:

1. Line 63: should be 'a corresponding interaction scenario' or 'corresponding interaction scenarios'. The 'a' and 'scenarios' disagree in plurality.

This has been corrected in the revised work.

2. How sensitive is the analysis to the chosen time scale?

The relevant timescale in our analysis is defined by the chosen sliding window. We provide a comprehensive sensitivity analysis for this window size in Fig S4. We find that a window size comparable to the short end of the inter-spike interval distribution captures the dynamics best. Empirically, as shown in Fig S6, the inference yields consistent relative trends across a reasonable range of window sizes, with a positive shift under larger window.

3. Line 227: Not sure what is meant by the phrase 'projection artifact.'

We thank the reviewer for pointing out this unclear terminology. We originally used the phrase "projection artifact" because the inferred marginal pairwise coupling (g_{ij})—while computed from pair statistics—can actually be computed from the triplet statistics. As shown in Appendix 3, g_{ij} depends on the total dwell times of different configurations of neurons i and k when neuron j is silent (the tau's), as well as various effective couplings from the triplet statistics: w_{ij}, w_{kj}, and w_{ij}' (effective coupling from i to j, conditioned on active k).

However, prompted by the reviewer's question, we re-examined our own terminology and realized that calling these discrepancies merely "artifacts" or "spurious links" was an oversimplification. During this revision, we have overhauled this concept and expanded Appendix 3 to provide a more detailed analytical formulation of this projection.

As detailed in the new Appendix 3, we explicitly derive how the marginal pairwise coupling (g_{ij}) is a state-dependent mixture of the conditional basal coupling (w_{ij}), the driven coupling (w_{ij}'), and the background drive from the third neuron (w_{kj}). Therefore, a discrepancy between g_{ij} and w_{ij} is an informative signature, rather than merely an indicator of statistical error. We show that $g_{ij} \sim w_{ij}$ only in the small firing rate limit of the neuron k. Beyond that limit, g_{ij} can significantly deviate from w_{ij} and instead reflects w_{ij}' (high firing

rate limit) or w_ij' + w_kj (high correlation limit of i and k), depending on the firing rates. The difference in g_ij and w_ij reflects the network modulation effects.

To reflect this deeper understanding, we have removed the term "projection artifact," and now concisely explain this phenomenon in the main text under the new heading "Network Modulation and Indirect Pathways" (Lines 235-241 in the revised manuscript), and direct mathematically inclined readers to the full derivation in Appendix 3.

4. Line 239: Where is the shared excitation indicated? Is that just the fact that units spiked from baseline, e.g. [0,0,0] to [1,0,0]?

We thank the reviewer for raising this point, which highlights the need for clearer definitions in the text. To clarify, the transition from [0,0,0] to [1,0,0] specifically measures the baseline intrinsic excitability (f_1), not shared excitation.

Instead, within the FLEC framework, the signature of unobserved common drive is captured by the contrast between the basal conditional coupling (w_ij) , computed when a third neuron k is silent, and the driven coupling (w_ij′), computed when neuron k is active.

In applying FLEC to the retina dataset, we found a consistent pattern (now illustrated in Fig. S8): the inferred basal couplings (w_ij) are predominantly negative. In contrast, the driven couplings (w_ij') frequently shift to positive values. Phenomenologically, we interpret the activation of neuron k as a statistical proxy indicating the presence of an external drive (e.g., the visual stimulus). A strong external stimulus would simultaneously drive the neurons, resulting in a positive effective coupling (w_ij′) that transiently overrides the baseline effective suppression (w_ij). We have expanded our discussion of this phenomenological signature in the revised manuscript (Lines 280-291).

Reviewer #2: This manuscript proposes a novel method for estimating functional connectivity directly from spiking activity. The authors evaluate the performance of the method using networks of LIF neurons and demonstrate that it performs favorably, particularly in strongly coupled regimes where conventional approaches such as generalized linear models (GLMs) and Granger causality (GC) may become unstable.

A notable strength of this work is that the proposed framework does not rely on a predefined statistical model such as a GLM. Instead, it directly maps network activity into a state space and characterizes neuronal interactions through state-transition rates. This is, to my knowledge, a novel and interesting approach. Developing alternative

methods for estimating neuronal connectivity is important for advancing our understanding of functional networks, and the present framework may become a useful addition to the growing set of analytical tools available for large-scale neural recordings.

The authors systematically evaluate the method under a variety of simulation conditions, including different connectivity patterns, connection strengths, and noise levels. These analyses provide valuable insight into the characteristics and limitations of the proposed approach. Overall, I view this work positively and believe it has the potential to make a meaningful contribution to the field.
That said, I believe several issues should be addressed or clarified before publication.

We thank the reviewer for their positive comments and constructive suggestions. We believe we have addressed all raised issues.

Major Comments

1. Scalability to large neuronal populations
The manuscript provides a thorough characterization of the method for small groups of neurons. However, it remains unclear whether the proposed framework is practical for large-scale networks (e.g., >100 neurons).
Although Figure 7 presents simulations involving 100 neurons, the analysis focuses only on neuronal triplets. Since the ultimate goal is network inference, it would be helpful if the authors could discuss in greater detail how the method scales when the number of neurons included in the analysis increases substantially (e.g., 10, 100, or more neurons). In particular, what are the practical computational and data requirements in such cases? As noted in the Discussion, the amount of data required appears to increase rapidly with the dimensionality of the state space. Are there strategies that could mitigate this problem? For example, the authors briefly suggest constructing larger networks from smaller motifs. Could hierarchical or motif-based approaches be used to efficiently identify candidate neuron groups and reduce the combinatorial complexity of the analysis? A more detailed discussion of the scalability and practical applicability of the method would strengthen the manuscript.

We thank the reviewer for raising this critical point. The reviewer correctly identifies both the fundamental challenge of our state-space approach and the theoretical strategy—hierarchical, motif-based subsampling. In this revision, we have expanded our discussion to formalize this strategy.

1. **Explicit Data Requirements and the "Exponential Wall":** To directly answer the question of practical data requirements, we have added a scaling argument in the revised text (Lines 209-217). A direct global application of FLEC to N neurons requires counting transitions across N2^N edges in the state space. As we note in the text, evaluating N=10 requires roughly 10^4 transition edges, but N=100 explodes to 10^32. Based on our

finite-sample analysis (new Fig. S7), reliable inference requires empirically about 100 counts per transition. Therefore, we have now stated that direct global FLEC inference becomes computationally and data prohibitive as network size increases.

2. **The Hierarchical Mitigation Strategy:** Because of this "exponential wall," we therefore motivate a motif-based approach. Prompted by the reviewer, we expanded our explanation of how marginal (g_ij) and conditional coupling (w_ij) serve as a perturbative expansion:

    1. The pairwise marginal coupling (g_ij) acts as the zeroth-order inference, absorbing all network influences.

    2. The triplet conditional coupling (w_ij) acts as the first-order correction, filtering out the specific influence of an observed neighbor by conditioning the third neuron's activity. In the revised Appendix 3, we analytically derive how w_ij and other triplet statistics mix to form g_ij.

3. **Robustness via Subsampling (In Vivo):** To demonstrate practical applicability, we added a new robustness analysis using the retinal data as suggested by the other referee (Fig. 7b and Fig. S8). Instead of fitting the entire network, we infer the effective coupling for a pair of neurons multiple times, each time conditioning on a *different* third neuron. The stability of the inferred inhibitory couplings across different conditional backgrounds shows that subsampling is a robust method for identifying effective couplings without requiring direct inference over the full N 2^N space.

As this work introduces the novel FLEC framework, our goal here is to establish the framework of this pair-to-triplet transition. As we now note in the Discussion, extending this logic to quadruplets (conditioning on two neighbors simultaneously) and developing automated candidate selection algorithms are the natural next steps for future studies.

2. Comparison with alternative connectivity measures
The manuscript compares the proposed method against Granger causality-based connectivity estimation. My understanding is that the implementation used here corresponds to a conventional form of Granger causality.
Have the authors considered comparing their method with variants specifically developed for spiking neural data (e.g., Kim et al., 2011, PLoS Computational Biology; Casile et al., 2021, PLoS Computational Biology)? It would be interesting to know whether such methods improve performance relative to the baseline GC implementation used in this study.

In addition, have the authors considered comparisons with Transfer Entropy-based approaches (e.g., Ito et al., 2011, PLoS ONE)? Since Transfer Entropy is often used to infer directed interactions from neural spike trains, including such a comparison—or

discussing the expected differences—would provide a broader context for evaluating the advantages and limitations of the proposed framework.

Thank you for pointing us to these related methods. Out of the three suggestions, we found Kim et al., 2011 most relevant and directly comparable. Casile et al., 2021 is an extension of Kim et al. with additional assumptions on cross-trial covariance. The Transfer Entropy approach (Ito et al., 2011) uses a similar state-transition paradigm; however, their original formulation uses *a priori* knowledge of synapse signs to distinguish excitatory and inhibitory interactions, making a direct comparison of signed coupling weights less straightforward.

Therefore, in the revised work we updated Figure 4 to include the "spiking Granger Causality" (spkGC) method following Kim et al., 2011. This approach adapts the original GC method for point processes. We find that spkGC performs better at strong weights compared to standard GC. FLEC nevertheless remains robust across a broader range of coupling strengths.

> *"The cosine angle between inferred and true weights as a function of the strength of networks with E-I balanced motif. The weights here are scalars bar{W} so that in each case the final weights are bar{W}W_{ij}, where W_{ij} is the E-I balanced motif with unit excitatory connection. The four colors indicate different methods: FLEC proposed in this work, Granger causality (GC) (Gerhard et al. 2013), generalized linear model (GLM) (Pillow et al. 2008), and spiking GC (spkGC) (Kim et al. 2011)."*

3. Interpretation of the application to retinal data
Regarding the application to salamander retinal ganglion cell recordings (p.12, line 237), additional biological interpretation would be valuable.
To what extent are the inferred connectivity patterns consistent with existing knowledge of salamander retinal circuitry? What types of retinal ganglion cells were recorded, and what biological significance should be attributed to the observed interaction patterns? Were the inferred relationships expected based on previous studies?

Furthermore, the manuscript highlights a specific three-neuron relationship.
Are there additional examples that support the generality of the findings?
Were similar interaction patterns observed across the recorded population, or was this example selected because it was particularly illustrative?

Additional examples or a more comprehensive summary of the inferred connectivity patterns would help readers assess the biological relevance of the results.

We thank the reviewer for pushing us to contextualize these findings and demonstrate their generality. Because this dataset (Palmer et al., 2015) consists of stimulus-driven recordings lacking ground-truth connectivity and cell-type labels, it precludes definitive anatomical claims. Nevertheless, we argue that the phenomenological signatures recovered by FLEC are consistent with known retinal physiology.

1) **Cell Types and Biological Significance:** The dataset does not provide morphological identities for the recorded retinal ganglion cells (RGCs). However, the dominant feature we inferred—widespread effective mutual inhibition—is consistent with known retinal circuitry. Lateral inhibition mediated by amacrine-cell networks is a well-established feature of retinal processing, while subsets of RGCs can also exhibit weaker electrical coupling through gap junctions. The inferred interactions therefore provide a plausible effective description of these suppressive and excitatory network influences. Importantly, we now explicitly clarify in the manuscript that these effective couplings should not be interpreted as direct anatomical synapses.

2) **Generality of the Findings and Additional Examples:** To confirm that the highlighted three-neuron relationship was not an isolated example, we expanded our analysis to a broader population of RGCs. We analyzed two additional triplet motifs, including neurons with high and low firing rates, and found qualitatively similar interaction patterns. We have included an additional representative triplet example in the new Fig. S5 of the Appendix.

3) **A Comprehensive Summary of Connectivity Patterns:** We have provided a comprehensive summary of the effective connectivity across the sampled population (updated Fig. 7 and new Fig. S8). We observe a consistent statistical phenomenology:

    - The basal conditional couplings (w) are predominantly negative, indicating widespread effective inhibition or refractoriness during baseline activity.

    - When analyzing transition rates under higher network activity (i.e., conditioning on the active state of a third neuron, w′), the effective interactions frequently shift toward positive values. This shift could reflect an unobserved common drive, such as the spatiotemporally correlated visual stimulus. A dynamic stimulus could simultaneously excite multiple RGCs, transiently overcoming the baseline effective inhibition and generating correlated spiking. We therefore interpret the difference between w and w′ as evidence of state-dependent network modulation, while avoiding a direct anatomical interpretation.

Minor Comments
p.12, lines 232–233: There appears to be a figure labeling error. Figure 5b should be Figure 5d, and Figure 5c should be Figure 5e.

Thank you for pointing out the error. We have corrected this.

Figure 6: The results may be easier to interpret if the inferred connectivity is also visualized as a network graph.

We explored network graph visualizations but found them less suited for conveying the specific quantitative insights of our revised manuscript. Because we now heavily emphasize the variance

and stability of effective couplings across different conditional backgrounds (as shown by the error bars in Fig 7b and Fig S8), a network graph would become visually cluttered, obscuring the quantitative error bars that are central to demonstrating our method's robustness. We therefore opted to retain the bar plots for quantitative transparency.

Figure 7: Since this figure addresses the performance of the method in a larger-scale network, it may fit more naturally within the Results section rather than the Discussion.

We have now moved the large-scale network inference into the Results section.

Figure 8b: Please clarify the meaning of the parameters W = 8 and W = 32.

This is the coupling strength parameter in the simulated synaptic current (Equation 7). We have now added a brief clarification in Figure 8's caption.
