# Supplementary material for: Inferring effective neuronal circuits via network flux counting
Source: PLoS Comput Biol. 2026 Sep 15;22(9):e1014763. doi: 10.1371/journal.pcbi.1014763 (PMC13592720; doi:10.1371/journal.pcbi.1014763)
Supplement: Peer review history

# Peer review: Decision Letter 0

**DOI:** 10.1371/journal.pcbi.1014763.r001

**Authors:** Hugues Berry, Ruben Sanchez-Romero

17 Jun 2026

PCOMPBIOL-D-26-01170

Inferring Effective Neuronal Circuits via Network Flux Counting

PLOS Computational Biology

Dear Dr. Chen,

Thank you for submitting your manuscript to PLOS Computational Biology. After careful consideration, we feel that it has merit but does not fully meet PLOS Computational Biology's publication criteria as it currently stands. Therefore, we invite you to submit a revised version of the manuscript that addresses the points raised during the review process.

Please submit your revised manuscript by Jul 17 2026 11:59PM. If you will need more time than this to complete your revisions, please reply to this message or contact the journal office at ploscompbiol@plos.org. When you're ready to submit your revision, log on to https://www.editorialmanager.com/pcompbiol/ and select the 'Submissions Needing Revision' folder to locate your manuscript file.

Please include the following items when submitting your revised manuscript:

* A letter that responds to each point raised by the editor and reviewer(s). You should upload this letter as a separate file labeled 'Response to Reviewers'. This file does not need to include responses to formatting updates and technical items listed in the 'Journal Requirements' section below.

* A marked-up copy of your manuscript that highlights changes made to the original version. You should upload this as a separate file labeled 'Revised Manuscript with Track Changes'.

* An unmarked version of your revised paper without tracked changes. You should upload this as a separate file labeled 'Manuscript'.

If you would like to make changes to your financial disclosure, competing interests statement, or data availability statement, please make these updates within the submission form at the time of resubmission. Guidelines for resubmitting your figure files are available below the reviewer comments at the end of this letter.

As the corresponding author, your ORCID iD is verified in the submission system and will appear in the published article. PLOS supports the use of ORCID, and we encourage all coauthors to register for an ORCID iD and use it as well. Please encourage your coauthors to verify their ORCID iD within the submission system before final acceptance, as unverified ORCID iDs will not appear in the published article. Only the individual author can complete the verification step; PLOS staff cannot verify ORCID iDs on behalf of authors.

We look forward to receiving your revised manuscript.

Kind regards,

Ruben Sanchez-Romero

Academic Editor

PLOS Computational Biology

Hugues Berry

Section Editor

PLOS Computational Biology

**Additional Editor Comments (if provided):**

Please see reviewers comments as they point to important revisions that need to be address to increase the validity and applicability of the proposed approach.

**Journal Requirements:**

If the reviewer comments include a recommendation to cite specific previously published works, please review and evaluate these publications to determine whether they are relevant and should be cited. There is no requirement to cite these works unless the editor has indicated otherwise.

1) We ask that a manuscript source file is provided at Revision. Please upload your manuscript file as a .doc, .docx, .rtf or .tex. If you are providing a .tex file, please upload it under the item type u2018LaTeX Source Fileu2019 and leave your .pdf version as the item type u2018Manuscriptu2019.

2) Please upload all main figures as separate Figure files in .tif or .eps format. For more information about how to convert and format your figure files please see our guidelines:

https://journals.plos.org/ploscompbiol/s/figures

3) Please upload a copy of Figure Fig 1-8 which you refer to in your text on pages 4, 7, 8, 10, 11, 12, and 13 and 14. Or, if the figure is no longer to be included as part of the submission please remove all reference to it within the text.

4) We notice that your supplementary Figures are included in the manuscript file. Please remove them and upload them with the file type 'Supporting Information'. Please ensure that each Supporting Information file has a legend listed in the manuscript after the references list.

5) Thank you for stating "All code to generate figures and analysis are in this repository: https://github.com/Kevin-Sean-Chen/MaxCal_network" However, we were unable to access the data using the information provided. Please review and update the Data Availability Statement to ensure that the underlying data are publicly accessible and that all accession numbers, links, or repository details are correct and functional.

6) We have amended your Competing Interest statement to comply with journal style. We kindly ask that you double check the statement and let us know if anything is incorrect.

**Reviewers' comments:**

Reviewer's Responses to Questions

**Comments to the Authors:**

**Please note here if the review is uploaded as an attachment.**

Reviewer #1: Chen and Yang introduce a novel approach to estimate the connectivity of a neural circuit from spiking activity. They treat each network state (e.g. [0,1,0]) as a node on a hypercube, with edges between those differing by a single change (e.g. compared with the previous example [0,0,0] or [1,1,0]). The rate at which those edges are traversed can be used to estimate the spontaneous rates of units, their effective coupling, and their nonlinear response functions.

The results are presented clearly with straightforward examples highlighting the advantages of this new approach. I don’t have any major theoretical criticisms of the work but do have some concerns regarding the scalability of this approach to large scale in vivo datasets and a potential degenerate situation that may yield false connections.

Major concerns:

1. While the authors raise the issue that this approach has poor scaling with the number of nodes, it is unclear how bad the problem is when going from 3 to 5 nodes, or 5 to 20, or 100. It would be informative to show the form this scaling takes, such as how the number of nodes and edges that need to be evaluated grow. In addition, it would seem as the number of units increases the sampling of specific transition rates gets lower, so how would the duration of a simulation/recording have to increase to account for this. Presumably this depends in part on the firing rate of the neurons and sliding window size of the neurons, so keep those constant to just demonstrate should be sufficient.

2. The suggested solution for large networks of neurons is to subsample pairs or triplets. The authors show that in most cases there is a positive cosine angle between the ground truth and the subsampled triplet. However, for data collected in vivo there is no ground truth, so instead it might be helpful to know what the variance would be in the estimated effective connectivity between a pair of units. To do this, you would choose a pair and then vary across multiple runs the 3rd unit in the triplet

3. There may be a degenerate case in this approach that would show a connection between two unconnected units. If two units both received input from the same third unit, but at slightly different lags, it would establish a fixed lagged relationship between their spiking. If the difference between the lags falls within the sliding window size, then that might show a connection between them. Would this pose a problem, and if not, can the authors show why? In cross-correlation analysis of point process data, this is usually dealt with by shuffling the data across trials (see https://pmc.ncbi.nlm.nih.gov/articles/PMC3856913/).

Minor concerns:

1. Line 63: should be 'a corresponding interaction scenario' or 'corresponding interaction scenarios'. The 'a' and 'scenarios' disagree in plurality.

2. How sensitive is the analysis to the chosen time scale?

3. Line 227: Not sure what is meant by the phrase ‘projection artifact.’

4. Line 239: Where is the shared excitation indicated? Is that just the fact that units spiked from baseline, e.g. [0,0,0] to [1,0,0]?

Reviewer #2: This manuscript proposes a novel method for estimating functional connectivity directly from spiking activity. The authors evaluate the performance of the method using networks of LIF neurons and demonstrate that it performs favorably, particularly in strongly coupled regimes where conventional approaches such as generalized linear models (GLMs) and Granger causality (GC) may become unstable.

A notable strength of this work is that the proposed framework does not rely on a predefined statistical model such as a GLM. Instead, it directly maps network activity into a state space and characterizes neuronal interactions through state-transition rates. This is, to my knowledge, a novel and interesting approach. Developing alternative methods for estimating neuronal connectivity is important for advancing our understanding of functional networks, and the present framework may become a useful addition to the growing set of analytical tools available for large-scale neural recordings.

The authors systematically evaluate the method under a variety of simulation conditions, including different connectivity patterns, connection strengths, and noise levels. These analyses provide valuable insight into the characteristics and limitations of the proposed approach. Overall, I view this work positively and believe it has the potential to make a meaningful contribution to the field.

That said, I believe several issues should be addressed or clarified before publication.

Major Comments

1. Scalability to large neuronal populations

The manuscript provides a thorough characterization of the method for small groups of neurons. However, it remains unclear whether the proposed framework is practical for large-scale networks (e.g., >100 neurons).

Although Figure 7 presents simulations involving 100 neurons, the analysis focuses only on neuronal triplets. Since the ultimate goal is network inference, it would be helpful if the authors could discuss in greater detail how the method scales when the number of neurons included in the analysis increases substantially (e.g., 10, 100, or more neurons). In particular, what are the practical computational and data requirements in such cases?

As noted in the Discussion, the amount of data required appears to increase rapidly with the dimensionality of the state space. Are there strategies that could mitigate this problem? For example, the authors briefly suggest constructing larger networks from smaller motifs. Could hierarchical or motif-based approaches be used to efficiently identify candidate neuron groups and reduce the combinatorial complexity of the analysis? A more detailed discussion of the scalability and practical applicability of the method would strengthen the manuscript.

2. Comparison with alternative connectivity measures

The manuscript compares the proposed method against Granger causality-based connectivity estimation. My understanding is that the implementation used here corresponds to a conventional form of Granger causality.

Have the authors considered comparing their method with variants specifically developed for spiking neural data (e.g., Kim et al., 2011, PLoS Computational Biology; Casile et al., 2021, PLoS Computational Biology)? It would be interesting to know whether such methods improve performance relative to the baseline GC implementation used in this study.

In addition, have the authors considered comparisons with Transfer Entropy-based approaches (e.g., Ito et al., 2011, PLoS ONE)? Since Transfer Entropy is often used to infer directed interactions from neural spike trains, including such a comparison—or discussing the expected differences—would provide a broader context for evaluating the advantages and limitations of the proposed framework.

3. Interpretation of the application to retinal data

Regarding the application to salamander retinal ganglion cell recordings (p.12, line 237), additional biological interpretation would be valuable.

To what extent are the inferred connectivity patterns consistent with existing knowledge of salamander retinal circuitry? What types of retinal ganglion cells were recorded, and what biological significance should be attributed to the observed interaction patterns? Were the inferred relationships expected based on previous studies?

Furthermore, the manuscript highlights a specific three-neuron relationship. Are there additional examples that support the generality of the findings? Were similar interaction patterns observed across the recorded population, or was this example selected because it was particularly illustrative? Additional examples or a more comprehensive summary of the inferred connectivity patterns would help readers assess the biological relevance of the results.

Minor Comments

p.12, lines 232–233: There appears to be a figure labeling error. Figure 5b should be Figure 5d, and Figure 5c should be Figure 5e.

Figure 6: The results may be easier to interpret if the inferred connectivity is also visualized as a network graph.

Figure 7: Since this figure addresses the performance of the method in a larger-scale network, it may fit more naturally within the Results section rather than the Discussion.

Figure 8b: Please clarify the meaning of the parameters W = 8 and W = 32.

**********

**Have the authors made all data and (if applicable) computational code underlying the findings in their manuscript fully available?**

The PLOS Data policy requires authors to make all data and code underlying the findings described in their manuscript fully available without restriction, with rare exception (please refer to the Data Availability Statement in the manuscript PDF file). The data and code should be provided as part of the manuscript or its supporting information, or deposited to a public repository. For example, in addition to summary statistics, the data points behind means, medians and variance measures should be available. If there are restrictions on publicly sharing data or code —e.g. participant privacy or use of data from a third party—those must be specified.

Reviewer #1: Yes

Reviewer #2: Yes

**********

PLOS authors have the option to publish the peer review history of their article (what does this mean?). If published, this will include your full peer review and any attached files.

If you choose “no”, your identity will remain anonymous but your review may still be made public.

**Do you want your identity to be public for this peer review?**  For information about this choice, including consent withdrawal, please see our Privacy Policy.

Reviewer #1: **Yes:** Drew Headley

Reviewer #2: No

[NOTE: If reviewer comments were submitted as an attachment file, they will be attached to this email and accessible via the submission site. Please log into your account, locate the manuscript record, and check for the action link "View Attachments". If this link does not appear, there are no attachment files.]

**Figure resubmission:**

**Reproducibility:**

To enhance the reproducibility of your results, we recommend that authors of applicable studies deposit laboratory protocols in protocols.io, where a protocol can be assigned its own identifier (DOI) such that it can be cited independently in the future. Additionally, PLOS ONE offers an option to publish peer-reviewed clinical study protocols. Read more information on sharing protocols at https://plos.org/protocols?utm_medium=editorial-email&utm_source=authorletters&utm_campaign=protocols

# Author comment: Author response to Decision Letter 1

**DOI:** 10.1371/journal.pcbi.1014763.r002

13 Aug 2026

Attachment Submitted filename: PCB_revision_response.pdf

# Peer review: Decision Letter 1

**DOI:** 10.1371/journal.pcbi.1014763.r003

**Authors:** Hugues Berry, Ruben Sanchez-Romero

26 Aug 2026

Dear Mr. Chen,

We are pleased to inform you that your manuscript 'Inferring Effective Neuronal Circuits via Network Flux Counting' has been provisionally accepted for publication in PLOS Computational Biology.

Before your manuscript can be formally accepted you will need to complete some formatting changes, which you will receive in a follow up email. A member of our team will be in touch with a set of requests.

Please note that your manuscript will not be scheduled for publication until you have made the required changes, so a swift response is appreciated.

IMPORTANT: The editorial review process is now complete. PLOS will only permit corrections to spelling, formatting or significant scientific errors from this point onwards. Requests for major changes, or any which affect the scientific understanding of your work, will cause delays to the publication date of your manuscript.

Should you, your institution's press office or the journal office choose to press release your paper, you will automatically be opted out of early publication. We ask that you notify us now if you or your institution is planning to press release the article. All press must be co-ordinated with PLOS.

Thank you again for supporting Open Access publishing; we are looking forward to publishing your work in PLOS Computational Biology.

Best regards,

Ruben Sanchez-Romero

Academic Editor

PLOS Computational Biology

Hugues Berry

Section Editor

PLOS Computational Biology

***********************************************************

Reviewer's Responses to Questions

**Comments to the Authors:**

**Please note here if the review is uploaded as an attachment.**

Reviewer #1: The authors have thoroughly addressed all the issues I raised.

Reviewer #2: The authors have adequately addressed my previous concerns in the revised manuscript. I am satisfied with the revisions and recommend acceptance.

**********

**Have the authors made all data and (if applicable) computational code underlying the findings in their manuscript fully available?**

The PLOS Data policy requires authors to make all data and code underlying the findings described in their manuscript fully available without restriction, with rare exception (please refer to the Data Availability Statement in the manuscript PDF file). The data and code should be provided as part of the manuscript or its supporting information, or deposited to a public repository. For example, in addition to summary statistics, the data points behind means, medians and variance measures should be available. If there are restrictions on publicly sharing data or code —e.g. participant privacy or use of data from a third party—those must be specified.

Reviewer #1: Yes

Reviewer #2: Yes

**********

PLOS authors have the option to publish the peer review history of their article (what does this mean?). If published, this will include your full peer review and any attached files.

If you choose “no”, your identity will remain anonymous but your review may still be made public.

**Do you want your identity to be public for this peer review?**  For information about this choice, including consent withdrawal, please see our Privacy Policy.

Reviewer #1: No

Reviewer #2: No

# Editor report: Acceptance letter

**DOI:** 10.1371/journal.pcbi.1014763.r004

**Authors:** Hugues Berry, Ruben Sanchez-Romero

PCOMPBIOL-D-26-01170R1

Inferring Effective Neuronal Circuits via Network Flux Counting

Dear Dr Chen,

I am pleased to inform you that your manuscript has been formally accepted for publication in PLOS Computational Biology. Your manuscript is now with our production department and you will be notified of the publication date in due course.

The corresponding author will soon be receiving a typeset proof for review, to ensure errors have not been introduced during production. Please review the PDF proof of your manuscript carefully, as this is the last chance to correct any errors. Please note that major changes, or those which affect the scientific understanding of the work, will likely cause delays to the publication date of your manuscript.

Soon after your final files are uploaded, unless you have opted out, the early version of your manuscript will be published online. The date of the early version will be your article's publication date. The final article will be published to the same URL, and all versions of the paper will be accessible to readers.

For Research, Software, and Methods articles, you will receive an invoice from PLOS for your publication fee after your manuscript has reached the completed accept phase. If you receive an email requesting payment before acceptance or for any other service, this may be a phishing scheme. Learn how to identify phishing emails and protect your accounts at https://explore.plos.org/phishing.

Thank you again for supporting PLOS Computational Biology and open-access publishing. We are looking forward to publishing your work!

With kind regards,

Janani Seenivasan

PLOS Computational Biology | Carlyle House, Carlyle Road, Cambridge CB4 3DN | United Kingdom ploscompbiol@plos.org | Phone +44 (0) 1223-442824 | ploscompbiol.org | @PLOSCompBiol
